# Estimating and accounting for tumor purity in the analysis of DNA methylation data from cancer studies

**DOI:** 10.1186/s13059-016-1143-5

**Published:** 2017-01-25

**Authors:** Xiaoqi Zheng, Naiqian Zhang, Hua-Jun Wu, Hao Wu

**Affiliations:** 1 Department of Mathematics, Shanghai Normal University, Shanghai, 200234 China; 2 Department of Mathematics, Weifang University, Weifang, Shandong 261061 China; 3 Department of Biostatistics and Computational Biology, Dana-Farber Cancer Institute and Harvard School of Public Health, Boston, MA 02215 USA; 4 Department of Biostatistics and Bioinformatics, Rollins School of Public Health, Emory University, 1518 Clifton Road, Atlanta, Georgia 30322 USA

**Keywords:** DNA methylation, Tumor purity, Differential methylation, Cancer epigenomics

## Abstract

We present a set of statistical methods for the analysis of DNA methylation microarray data, which account for tumor purity. These methods are an extension of our previously developed method for purity estimation; our updated method is flexible, efficient, and does not require data from reference samples or matched normal controls. We also present a method for incorporating purity information for differential methylation analysis. In addition, we propose a control-free differential methylation calling method when normal controls are not available. Extensive analyses of TCGA data demonstrate that our methods provide accurate results. All methods are implemented in InfiniumPurify.

**Electronic supplementary material:**

The online version of this article (doi:10.1186/s13059-016-1143-5) contains supplementary material, which is available to authorized users.

## Background

The role of DNA methylation in cancer has been studied extensively over several decades, in the hope of identifying diagnostic biomarkers and therapeutic targets [1–3]. Recent developments in high-throughput technologies, such as Illumina Infinium 450 k microarray [4] and bisulfite sequencing [5, 6], have revolutionized the cancer epigenomics research. Enormous amounts of data have been generated from these platforms, for example, by large international consortiums like The Cancer Genome Atlas (TCGA) or the International Cancer Genome Consortium (ICGC). Analysis results from these data have greatly advanced our knowledge in cancer epigenomics and provide valuable targets for drug development [7–10].

One important problem in cancer genomics or epigenomics research, especially from high-throughput technologies, is that the solid tumor tissues obtained from clinical practice are highly heterogeneous. They are always mixtures of cancer cells, adjacent normal tissues, stromal, and infiltrating immune cells. In high-throughput DNA methylation experiments, the whole tumor sample is processed to extract DNA from all cells and then the methylation levels are profiled. Thus, the measurements are in fact mixed signals from different cell types. If not correctly accounted for, such sample mixture could bias the downstream data analyses such as differential methylation and sample clustering, since it increases the within group variation and masks the true biological signal [11].

The sample mixture problem in cancer study was identified a while ago. Estimating the “tumor purity”, or the percentage of cancer cells in a solid tumor sample, has been an active research topic [12, 13]. Experimentally determining the cancer purity is possible through cell sorting-based technology such as Fluorescent-Activated Cell Sorting (FACS) [14] or Magnetic-Activated Cell Sorting (MACS) [15]. These methods, however, are laborious and expensive thus cannot be applied to large-scale studies. Fortunately, it was discovered that *in silico* estimation of tumor purity from high-throughput data is feasible because of the marked differences in genomics and epigenomics profiles between cancer and normal cells. These differences, including differential gene expression, differential methylation, and different mutation and copy number variation patterns, can be used as predictors to estimate tumor purity. A number of statistical methods have been developed for the purpose, based on genetic variants (single nucleotide polymorphism (SNP) or copy number variation) [12, 16], gene expression [13, 17], or DNA methylation data [18–20]. Wang et al. provide a comprehensive review for available purity estimation methods [21]. Many of these methods share a similar approach that data from tumor samples are modeled by a mixture distribution where tumor purity is a latent parameter and the purity estimation is performed by maximizing the data likelihood. Method implemented in the RefFreeEWAS R package provides “reference-free” deconvolution [20], which does not require data from purified samples. The problem is similar to the blind signal separation (BSS) in signal-processing field [22, 23] and the deconvolution is achieved by non-negative matrix factorization.

In spite of the successes, there are a number of limitations in these purity estimation methods. First, many methods require data from “reference samples” (the pure cancer/normal samples) as predictors in a linear model framework to estimate tumor purity. The reference data could be difficult or expensive to obtain in practice. The reference-free method, such as RefFreeEWAS, requires data from a large number of tumor samples so that the matrix factorization can be performed stably. This poses difficulties for a smaller-scale study. Moreover, several methods require data from normal controls so that differential expression/methylation can be identified and used as predictors [11, 24], which are also not easy to obtain sometimes. Finally, some methods are based on complicated statistical modeling that requires substantial amount of computation [12, 25].

Differential methylation (DM) analysis in cancer-normal comparison is an important task in cancer epigenomics research. The differentially methylated CpG sites (DMCs) or regions (DMRs) identified from such analysis can be further associated with somatic mutations [26] or gene expression regulation [27] to enhance our understanding of cancer etiology. Moreover, the DMCs/DMRs could potentially serve as diagnostic biomarkers or therapeutic targets [28–31]. Current methods for DM analysis usually ignore the purity information and treat data from tumor samples as independent biological replicates [32–40]. Such an approach is undesirable because the data from tumor samples do not follow the same distribution due to differences in the purity. Ignoring the purity could lead to biased, even erroneous results. A closely related problem is the adjustment of cell composition from heterogeneous samples such as blood or brain in the epigenome-wide association study (EWAS) [11, 41–43]. Such a problem assumes multiple components in the mixture and that the mixing proportion can be related to experimental conditions. The goal is to eliminate the effect of differences in mixing proportions and detect changes caused by experimental factor of interest. The problem is very similar to earlier works on removing hidden confounding factors (such as batch effect) [44, 45] and a few methods were developed based on different methods such as singular value decomposition [42] or linear mixed model [43]. The goal of this problem, however, is fundamentally different from the DM analysis in cancer-normal comparison, assuming both case and control samples are mixtures of two types of cells A and B and one wants to detect methylation changes between cases and controls. The EWAS tries to find sites that both A and B change (in the same direction) between case and control, adjusting for potential differences in mixing proportions, whereas the DM analysis is to find the difference between A and B. Due to this reason, the methods developed for EWAS are not directly applicable for cancer-normal comparison. To the best of our knowledge, the method for DM analysis with consideration of tumor purity is not yet available. There are some practices to account for purity in differential expression (DE) analysis [46] by adding purities as a covariate in the linear model. As we will show, the purity should have a multiplicative effect instead of an additive effect. In addition, the normal controls are sometimes difficult or expensive to obtain in a cancer study, for example among all available 450 k methylation array data in TCGA, 17 of 32 cancer types have less than five normal samples, while ten of them are completely absent of normal samples. When normal controls are unavailable, it seems the DMCs/DMRs between cancer and normal cannot be detected.

With the continuous cost reduction of technology, large-scale, population level methylation studies have become increasingly prevalent for different types of cancers. The rapid accumulation of data requires a better method for analysis. In this work, we make three important contributions to the field of DNA methylation analysis in cancer. First, we extend our previously developed method for estimating tumor purity from Illumina Infinium 450 k methylation microarray data. The updated purity estimation procedure does not require data from reference samples, matched normal controls, or purity estimated from other tools. The algorithm is extremely simple, intuitive, and computationally efficient, yet it provides results highly consistent with methods based on other data types. Second, we develop a statistical method, based on a linear model, to perform DM analysis for 450 k data with the consideration of tumor purity. Parameters are estimated using a generalized least square and hypothesis testing for DMC is achieved by the Wald test. Finally, we develop a method for detecting DMCs when normal control data are absent. The method draws inferences of DMCs based on the correlation between methylation and purity levels. We show by extensive real data analyses that the proposed methods are sensitive, accurate, and computationally efficient. All proposed methods are implemented in the latest version of InfiniumPurify, which is freely available at https://zenodo.org/record/200214.

## Results

### The newly updated method for purity estimation

The previously developed InfiniumPurify for purity estimation [18] is based on an important observation from the 450 k methylation data: the number of probes with intermediate methylation level is significantly greater in tumors compared to normal samples. Many of the intermediate methylated CpG sites are the result of sample mixtures and contain information of the mixing proportion (tumor purity). InfiniumPurify first identifies a number of informative differentially methylated CpG sites (iDMCs) from cancer-normal comparisons and then estimates purity from the probability density of methylation levels of iDMCs. An important drawback of the previous version of InfiniumPurify is that the selection of iDMCs requires a number of cancer and normal samples. For cancer types without or only having a few normal samples, such as ovarian carcinoma (without a normal sample) or glioblastoma (only one normal sample) from TCGA, InfiniumPurify would fail or has not enough statistical power to find reliable iDMCs. Our previous method therefore was only able to provide estimated tumor purities for nine cancer types in TCGA. This greatly limits the application of InfiniumPurify in smaller scale studies or on new cancer types.

**Fig. 1:**
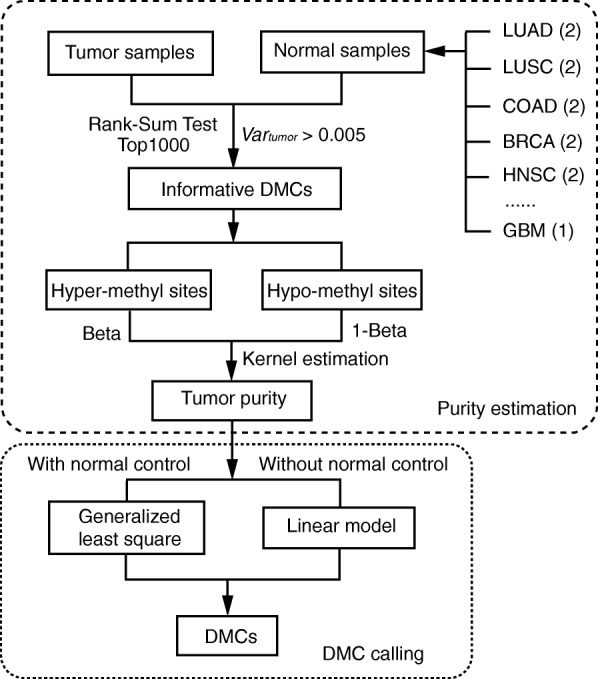
A *flowchart* to illustrate the InfiniumPurify algorithm, including purity estimation and DM calling procedures

We obtained all 450 k methylation data from TCGA (including 8830 tumor samples and 703 normal samples for 32 cancer types) to study the effect of iDMC selection and purity estimation. We found that it is possible to use a group of “universal” normal samples to obtain iDMCs and then apply them on purity estimation for different cancers. We redesigned the purity estimation algorithm, which can be applied to data without normal controls or replicates. The essence of the newly updated method is to combine normal samples from different tissue types, construct a panel of normal methylomes, and then detect iDMCs for each cancer type using this panel for downstream purity estimation. Another important improvement of current version of InfiniumPurify is that it does not rely on ABSOLUTE to calibrate the estimation. Therefore, all purity results in this paper are from 450 k methylation array data alone. The comparison with existing methods shows that tumor purities using universal normal samples are comparable with previous version, even better for cancer types with only small number of normal samples. The algorithm of updated InfiniumPurify is illustrated in Fig. 1, and is detailed in the “Methods” section.

### Genomic locations of iDMCs

We carefully investigated the genomic locations of the iDMCs. Taking the average across all cancer types, 22% of the iDMCs are located at the transcriptional start site (TSS), 3% are at the transcriptional end site (TES), 11% at the exonic regions, 32% at the intronic regions, and 31% at the intergenic regions. Compared with all CpG sites on the 450 k array, these iDMCs are relatively depleted at gene promoter regions and enriched at intergenic regions. This indicates that the iDMCs are more likely to appear in the less important regions. Moreover, the iDMCs are rather dispersed along the genome (the top 1000 iDMCs are located in 432 genes on average). The spatial diversity of the iDMC is a desirable feature because the purity estimation will not be overly influenced by the differential methylation by a few genes. Finally, overlaps of iDMCs from different cancer types are rather low: the average pairwise overlap is only 2.8%. These results demonstrate the cancer type specificity of iDMCs, thus it is necessary to obtain a set of iDMCs for each cancer. We also associate each iDMC to a gene if it is located within 3000 bps to the gene. On average, iDMCs are located in 432 genes (Additional file 1: Material Section S1). Most (89%) of the iDMC-bearing genes contain only one or two iDMCs, thus the locations of iDMCs are rather dispersed. The spatial diversity is desirable and potentially more robust, because the purity estimation result will not be overly influenced by the differential methylation by a few genes. More detailed description of the iDMC location is provided in Additional file 1: Material Section S1.

### Tumor purity estimation results from TCGA data

#### Overall purity estimates and their correlation with other methods

**Fig. 2:**
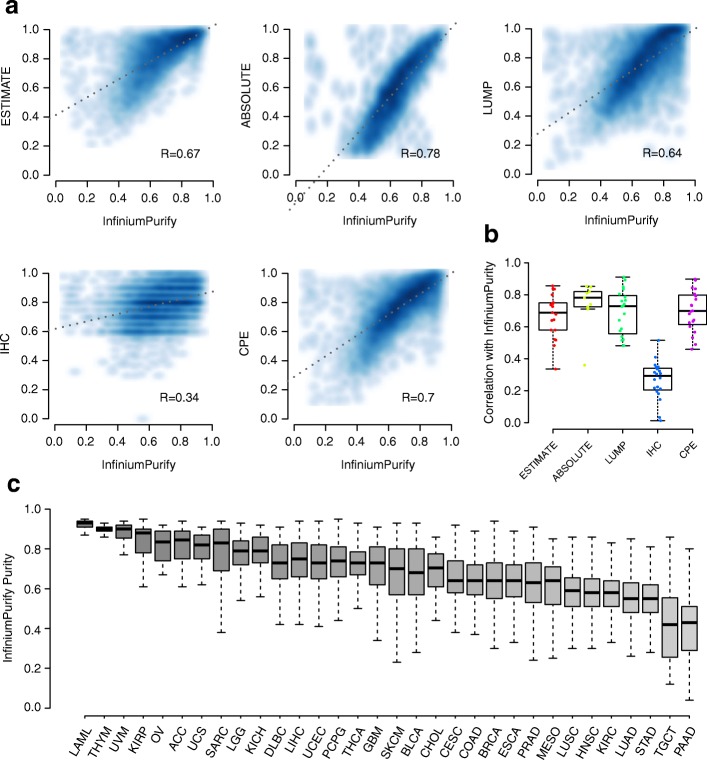
Purity estimates from TCGA data. **a** *Scatter plots* showing comparison of purities for all TCGA tumor samples from InfiniumPurify with ESTIMATE, ABSOLUTE, LUMP, IHC, and CPE, respectively. **b** Correlations between InfiniumPurify and other estimates for all TCGA cancer types. **c** Distribution of estimated tumor purities from InfiniumPurify for all TCGA cancer types

We then applied InfiniumPurify to all TCGA tumor samples whenever the 450 k data are available (8830 samples from 32 cancer types). To compare our estimated purities with other methods, we obtained purity estimates for all cancer samples from [46], based on different methods including ABSOLUTE [12], ESTIMATE [13], a consensus measurement of purity estimation (CPE) [46], image analysis of hematoxylin and eosin stain slides (IHC) [46], and non-methylation of immune-specific CpG sites (LUMP) [46]. Overall, the InfiniumPurify estimates have good correlations with these except IHC. Figure 2a shows the scatter plots of estimated purities from InfiniumPurify versus other methods for all samples in all cancer types. InfiniumPurify estimates have the highest Pearson’s correlation with ABSOLUTE (Pearson’s correlation 0.78) and the lowest correlation with IHC (Pearson’s correlation 0.34). For each individual cancer type, the correlations between InfiniumPurify and other estimates are also high (Additional file 2: Figures S1–S5), showing that the good overall consistence is not due to cancer type bias or a few outliers. Figure 2b summarizes such correlations from different cancer types. A barplot of these correlations (with cancer names) is also provided in Additional file 2: Figure S6. The correlations are mostly high except for IHC, which is consistent with the findings in [46]. It is because IHC is based on image analysis and the data are substantially different from other methods. Overall, we find consistently high correlation between purity estimates from InfiniumPurify and other methods.

We further looked at the distributions of estimated purities of individuals from different cancer types, shown in Fig. 2c. Overall, LAML (acute myeloid leukemia) has the highest purity, followed by THYM (thymoma), both of which have very small variance in different patients. On the other hand, PAAD (pancreatic adenocarcinoma) and TGCT (testicular germ cell tumor) have the lowest average purities, indicating that the small size of these tumors causes difficulty and variability in collecting the tumor samples in operation. A few other examples with low average purities are LUSC (lung squamous cell carcinoma), HNSC (head and neck squamous cell carcinoma), KIRC (kidney renal clear cell carcinoma), and LUAD (lung adenocarcinoma), which are also predicted as low consensus purities by [46]. In general, this result is consistent with the one reported in Fig. 1b of [46]. However, we were able to generate purity estimates for more cancer types due to the wider availability of DNA methylation data.

### Effect of iDMC selection on purity estimation

We performed several analyses to investigate the effect of iDMCs selection on purity estimation. First, we investigated the effect of number of iDMCs on purity estimation. We selected different numbers of iDMCs (top N by rank-sum test statistics) and evaluated the purity estimates by their correlation with ABSOLUTE purities. An example for lung adenocarcinoma LUAD is provided in Additional file 3: Table S1. In general, the results are rather stable: the correlations with ABSOLUTE are similar when selecting 50–5000 iDMCs. The correlations become lower when using more iDMCs. For examples, the correlation is decreased to 0.076 if selecting 30,000 iDMCs. This is because using too many iDMC brings extra noise to the estimation. Overall, the purity estimation procedure is reasonably stable to the number of iDMC used and we recommend using top 1000 iDMCs.

We also explored the possibility of using normal blood samples as controls in iDMC detection, since the blood samples are much easier to collect in clinical practice. We obtained DNA methylation data for whole blood of 656 human samples aged 19–101 years [47] and randomly selected 50 samples as normal controls for iDMC and purity estimation. As shown in Additional file 2: Figure S7, purities estimated from using blood controls are highly correlated with those by universal normal controls for most cancer types. Their correlations with estimates from other methods are also comparable with those from using universal normal controls (Additional file 2: Figure S8). Nevertheless, we still observed a few cancer types, such as DLBC, LAML, and THYM, whose predicted purities by blood control are poorly (or even negatively) correlated with our previous estimation by universal normal controls. One likely explanation for this phenomenon is that these tumor tissues may have very distinct methylation profiles from blood tissue, so the obtained iDMCs are mostly blood tissue-specific, not necessarily associated with differential methylation between tumor and normal. Thus, we want to emphasize that the accuracy of the purity estimation result could depend on tumor types and the users should use blood samples as controls with caution. It is also our future work to find more reliable iDMCs for purity estimation, especially in the blood control scenario. Overall, these results demonstrate that the proposed purity estimation procedure is robust and not affected much by iDMC selection and that using blood control to identify iDMC for purity estimation is possible.

We also conducted a tenfold cross-validation in iDMC identification and purity estimation. In detail, all tumor samples from specific cancer types are divided into roughly ten equal groups. Each group is iteratively served as test set, where iDMCs are obtained from remaining nine groups based on the above previous procedures. Results show that the tumor purities estimated from tenfold cross-validation are almost perfectly correlated with that by the whole dataset (Additional file 2: Figure S9).

Taken together, these results strongly validate the robustness and good performance of our purity estimation method, as well as other genomics data-based methods. They also demonstrate that different genomics and epigenomics signals provide rather consistent information on tumor purity. Different purity estimation procedures are based on different types of genomic data and from completely different algorithms, yet produce highly consistent results. This provides researchers more confidence in these estimates. However, it is worth mentioning that we do not claim superiority over any other purity estimation method since it is difficult to evaluate the performance without gold standards. We claim that: (1) the high correlation among different methods and from different data types provide validation to each other; and (2) InfiniumPurify is the first tool to provide purity estimation from methylation data, which is useful and complementary to other methods.

With these results, we will take the estimated purities as known constants and develop methods for differential methylation analysis with consideration of purities, detailed in later sections.

### Correlation between methylation levels and tumor purities

**Fig. 3:**
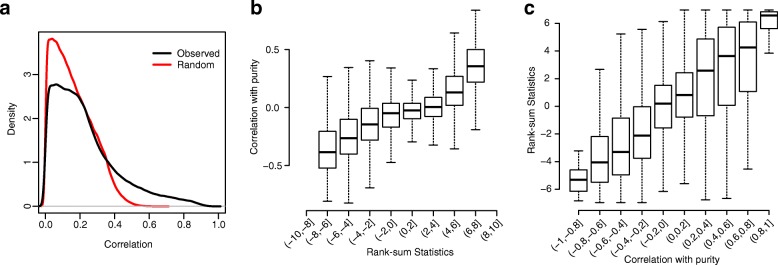
Correlations between tumor purity and methylation levels are high for DMCs. **a** *Distribution densities* of correlations between tumor purity and methylation levels for all CpG sites, from observed and randomly sampled data, based on LUAD data. **b** *Boxplots* of correlations, stratified by rank-sum test statistics. **c** *Boxplots* of rank-sum test statistics, stratified by correlations

Through extensive exploration of the TCGA data, we observed that at differentially methylated CpG sites (DMCs), methylation levels in tumors are highly correlated with tumor purities. To be specific, given a set of 450 k data from a number of tumor samples, each has a purity value (estimated from InfiniumPurify). For each CpG site, we computed the correlation between the beta values and the purities across samples. From there we obtained 450 k correlation values for this dataset, each for a CpG site. We also randomly shuffled the sample labels, computed the correlations, and used these numbers as controls for comparison purposes. Figure 3a plots the distributions of observed and random correlations for LUAD samples. It shows that the distribution of observed correlations has a longer right tail, indicating that there are many more CpG sites with high correlation with purities.

Next, we looked at the relationship of such correlations and differential methylation. We applied a Wilcoxon rank-sum test on all CpG sites to obtain test statistics. The CpG sites are then categorized into different groups by the test statistics and the distributions of correlations in each category are shown in the boxplots in Fig. 3b. It clearly shows that CpG sites with greater test statistics tend to have beta values more correlated with purities. Figure 3c shows the stratified boxplot in another direction: CpG sites are categorized by correlations and distributions of test statistics are displayed for each category. Similarly, CpG sites with higher correlations tend to have greater test statistics, hence are more likely to be differentially methylated.

We performed the above analysis for all available cancer types and observed the same phenomenon. These results indicate an important finding: DMCs tend to have beta values with greater correlation with purities. This observation is expected because only when the methylation levels are markedly different between cancer and normal samples, the mixed signal will correlate with mixing proportion. For CpG sites showing similar levels of methylation in cancer and normal samples, the mixed signal will be close to a constant regardless of the purity. This observation is an important foundation for our development of the DM calling method with consideration of purity.

### Differential methylation analysis with normal control

When normal control data are available, we developed a statistical method to call DMC with consideration of tumor purity. The performance of the method is demonstrated in both simulation studies and real data analyses.

#### Simulation

We performed extensive simulation to compare the performances of DM calling from different methods. We used the LUAD as a template to generate data so that the simulated data matched the real data characteristics. We conducted simulations for various scenarios under different sample sizes and signal-to-noise ratios. A detailed description of the simulation is provided in Additional file 1: Materials Section S2. The results show that under all simulation settings, InfiniumPurify provides the best performance. These simulation results demonstrate the robustness and accuracy of InfiniumPurify in DM calling in cancer study when tumor purity is a concern.

#### TCGA data results

We further applied the proposed DM calling method to all TCGA data whenever the 450 k data were available. We compared the DMC calling results with minfi [40], arguably the most widely used package for 450 k data analysis, and RefFreeEWAS, which considers cell composition in DM calling. We ran minfi using default parameters and specified K = 2 in RefFreeEWAS, corresponding to two components (cancer and normal) in the cell mixture. We want to point out that the comparison is not completely fair, since minfi does not consider purity and RefFreeEWAS is not designed for cancer-normal comparison (as discussed in the “Background” section). However, because there is currently no DM calling method accounting for purity, the results presented in this section simply demonstrate that the DM calling results can be significantly improved with proper consideration of purity. Even though there are a number of other DM calling tools for 450 k data [32–39, 48], none of them considers tumor purity so we expect they provide results similar to minfi. Due to this reason, those methods are not included in the comparison.

**Fig. 4:**
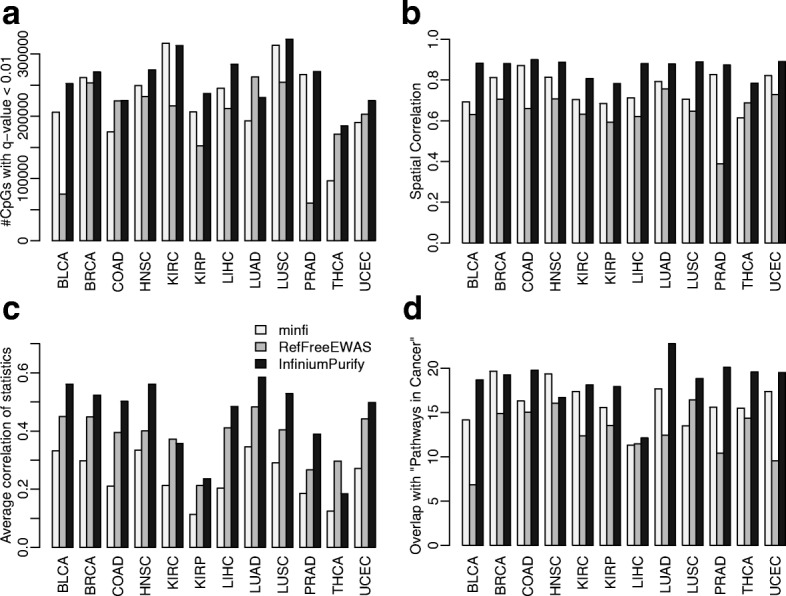
Differential methylation analysis results, with normal control. **a** Numbers of differential methylated CpG sites (FDR < 0.01). **b** Spatial correlations among test statistics from nearby CpG sites. **c** Average pan-cancer correlation of test statistics. **d** Enrichment *p* values for top 1000 differentially methylated genes within “PATHWAY-IN-CANCER” from KEGG

First, we examined the sensitivity in DM calling. Figure 4a shows the number of significant (defined as false discovery rate (FDR) < 0.01) DMCs detected for all cancer types whenever data are available. The proposed method detects the most DMCs in almost all datasets, demonstrating higher sensitivity. This makes sense because with the consideration of purity, the within group variance is reduced among the cancer samples, thus leading to a more powerful statistical test. The gain in sensitivity could be significant, for example, the number of DMCs detected in THCA (thyroid carcinoma) is almost doubled compared to minfi. On average, there are over 20% more DMCs detected from the proposed method compared to other methods. We also investigated the overlaps of DMCs called from different methods, shown by Venn diagrams in Additional file 2: Figure S10. It is shown that DMCs called from all three different methods have rather significant overlap for all tested cancer types, especially between InfiniumPurify and minfi.

We compared the absolute methylation differences for InfiniumPurify exclusive, minfi exclusive, and common DMCs from BRCA data. As shown in Additional file 2: Figure S11, InfiniumPurify exclusive DMCs show a much higher methylation difference between matched tumor and normal samples than minfi exclusive DMCs. This is because the InfiniumPurify exclusive DMCs have large within-group variances, caused by the tumor purities, thus they cannot be detected by minfi. After correcting for purity, the within-group variances are reduced and these sites will be called as DMC. This further illustrates the importance of purity correction in DM calling.

Next, we looked at the spatial correlations of test statistics from different methods. For each cancer type, we first selected pairs of CpG sites with distances less than 50 base pairs and computed the Pearson’s correlation of their test statistics. It was known that methylation levels have strong spatial correlation [49], that is, the nearby CpG sites usually have similar methylation levels. Therefore, the differential methylation statuses are likely to be similar among nearby CpG sites and this is the reasoning of grouping DMCs into DMRs in whole genome methylation data. Thus, we argue that a better DMC calling method should produce test statistics with stronger spatial correlation. Figure 4b compares the spatial correlations in test statistics from the three methods and the proposed method provides the highest correlation for all cancer types. This indicates that by accounting for purity in DM detection, the DM status from nearby CpG sites become more similar.

We further looked at the correlations among test statistics from different types of cancers. Even though different cancer types have distinct etiologies, they also share many commonalities, such as the hyper-methylation in CpG islands and genic regions and global hypo-methylation in whole genomes especially for highly and moderately repeated DNA sequences [50]. Hence, we believe that there are many shared epigenetics dynamics in different cancers and expect that the test statistics are well correlated across different cancer types. Figure 4c shows, for each cancer type, the average correlations in test statistics with other cancers. All inter-cancer correlations from three methods are shown in Additional file 2: Figure S12. Overall the test statistics from the proposed method have a stronger correlation, again suggesting that the results are more consistent.

Finally, we looked at the biological implications of the DM calling results. We first identify the top 1000 genes (termed as DMGs) with most DMCs by different methods. Then DMCs mapped to these genes are input to gometh function in missMethyl package [51] to test their enrichments with “PATHWAYS_IN_CANCER” from KEGG [52]. Compared to the simple Chi-square test, gometh function adjusts the bias from different numbers of probes on different genes, thus provide more objective results. Figure 4d shows the -log10 of the *p* values for the enrichment of DMGs in “PATHWAYS_IN_CANCER,” which contains 328 genes involved in all cancer types. The *p* values are much smaller from the proposed method, indicating stronger enrichment. We further examined the enrichment of DMGs in pathways related to different cancer types (Additional file 2: Figure S13). To be specific, we looked at the enrichment of DMGs from COAD (colon adenocarcinoma) in the COLORECTAL_CANCER pathway, UCEC (uterine corpus endometrial carcinoma) in the ENDOMETRIAL_CANCER pathway, PRAD (prostate adenocarcinoma) in the PROSTATE_CANCER pathway, THCA in the THYROID_CANCER pathway, BLCA (bladder urothelial carcinoma) in the BLADDER_CANCER pathway, and LUAD in the NON_SMALL_CELL_LUNG_CANCER pathway. Again, the enrichments are in general stronger from the proposed method. These results support that the proposed method generates more biologically meaningful results.

To better understand the differences in DM calling results from the proposed and other methods, we explored the raw data of CpG sites with substantial discrepancies in test results from the InfiniumPurify and minfi. Additional file 2: Figure S14 shows several examples of such CpG sites. These CpG sites are not statistically significant from minfi, mainly because of the large variance in the cancer group. However, the middle panel shows the scatter plot of beta value versus purities, indicating that the large within group variance is mostly caused by the variation in purities from different samples. After correcting the purity effect, as shown in the right panel, the adjusted beta values become higher and the means between two groups are visibly different now. This leads to a very significant test result and tiny *p* values (*p* < 1e-20). These examples illustrate the importance of correcting purity in the DM calling procedure.

Taken together, the results presented in this section show that the proposed DM calling method is more sensitive, accurate, and provides more biologically interpretable results compared with existing methods.

### Differential methylation analysis without normal control

**Fig. 5:**
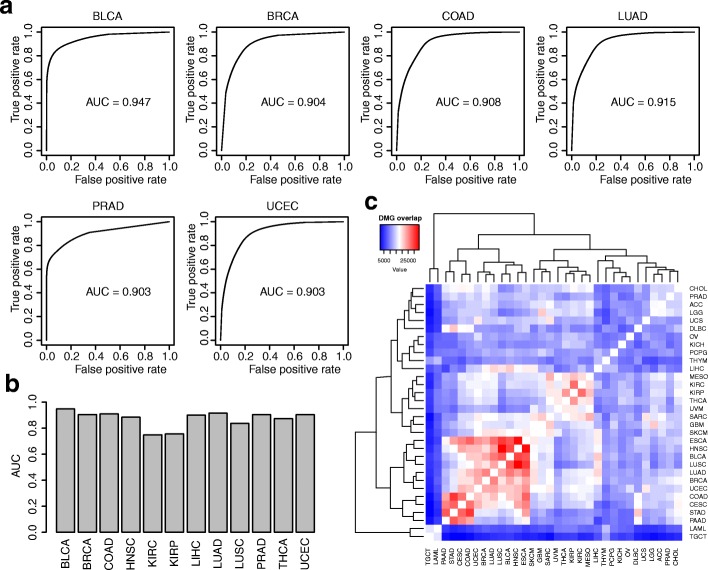
Differential methylation analysis, without normal control. **a** Examples of ROC curves from InfiniumPurify control-free DMC calling model, where results from tumor-normal comparison are treated as gold standard. **b** AUCs for selected cancer types in TCGA. **c** *Heatmap* showing overlaps of the top 50,000 DMCs among different cancer types

Taking advantage of the observation that methylation levels for DMCs tend to have higher correlation with purities, we developed a method to call DMCs without normal control. We then applied the method to all TCGA samples to call DMCs without using the data from normal samples. The DMCs called with control data are used as gold standard to benchmark these results. We generated receiver operating characteristic (ROC) curves for the results from all cancer types (Figure 5a and Additional file 2: Figure S15). The barplot of areas under the curve (AUCs) of all ROC curves is shown in Fig. 5b. Overall the results are satisfactory with the average AUCs being 0.873. Results from most cancer types are fairly accurate, for example, BLCA, BRCA, COAD, LUAD, PRAD, and UCEC all have AUC over 0.9. Results from KIRC and KIRP (kidney renal papillary cell carcinoma) are relatively worse with AUCs around 0.75. Another possible solution in DM calling when matched control samples are unavailable is to use a universal set of normal samples. We conducted such analysis and found that it produces slightly worse results than the control-free method. The detailed analysis is provided in Additional file 1: Materials Section S3.

We further looked at the accuracies of control-free DM calling from top ranked DMCs. This is sometimes more important than all the ROC curves for many high-throughput experiments, since the top ranked features are often of more interests and have undergone more investigation. Additional file 2: Figure S16 shows the true discovery rates (TDRs) for the top 50,000 CpG sites for a number of cancers. The accuracies are very high: on average about 95%. Even for KIRC and KIRP which have poor AUCs, the accuracies are fairly high at 87% and 83%, respectively.

We further performed pan-cancer analysis on the DMCs. Figure 5c shows the overlaps of top 50,000 DMCs across all cancer types. Generally, we found that the tumors originating from the same and nearby organs (such as lung, kidney, and uterine tumors) or originating from similar tissue/cell type (such as adenocarcinoma and sarcoma) share similar DMCs. For instance, the tumors originating from the upper respiratory tract form a clear differential methylation cluster, including ESCA (esophageal carcinoma), HNSC, LUSC, and LUAD. Two kidney cancers, KIRC and KIRP share a lot of DMCs. For the glioma, although GBM (glioblastoma multiforme) is clustered far from LGG (brain lower grade glioma), it shares the highest DMCs with the latter. Interestingly, UCS (uterine carcinosarcoma) shares more DMCs with the same organ as the tumor UCEC and also shares DMCs with SARC (sarcoma) and both originate from similar cell types. Note that such analysis is not feasible using the traditional method because many cancers do not have data from corresponding control samples. With our proposed method, more biological results can be obtained.

It is important to point out that the proposed control-free DM calling method requires relatively larger sample size (e.g. >20) and that the purities among samples need to be dispersed enough. The results could also be affected by the signal-to-noise ratios in the data (the ratios of inter-group and intra-group variations). For some cancer types, control-free DM calling could provide undesirable results. Nevertheless, the control-free DM calling results are satisfactory overall. We want to emphasize that one should profile the normal controls whenever possible if differential methylation is a major research interest. However, when normal control data are absent due to clinical or economic reasons, we provide a viable solution for DM calling.

## Discussion

Existing methods for tumor purify estimation are mainly based on gene expression or copy number data from either SNP array or high-throughput DNA sequencing. InfiniumPurify is the first method to provide purity estimation from DNA methylation microarray data. We want to emphasize that in cancer studies, the genetic or epigenetic data (genetic variants, gene expression, DNA methylation, etc.) are not specifically generated to measure cancer purity: those experiments are performed to study different aspects of cancer. Thus, it is important to be able to estimate purity from all types of data. If the purity could only be estimated from copy number data, then in an EWAS study where only DNA methylation data are available, one would not be able to estimate purity. From this perspective, it is equally important and useful to have purity estimation methods from different data types. In addition, DNA methylation is deemed to be more stable than gene expression, so it is potentially more accurate in purity estimation problem. Although copy number alteration is a characteristic of cancer cells and is also less variable than gene expression, cancer cells often have aberrant overall ploidy number compared with normal cells, which greatly affects copy number-based purity estimates. For example, ABSOLUTE needs a user selected tumor ploidy in determination of the optimal likelihood. Due to these reasons, tumor purity estimated from DNA methylation data could be more stable or at least plays a complementary role to exiting tumor estimation approaches.

There are some reports about the intermediate methylated (IM) sites and the methylation heterogeneity in cancer [53–57]. We want to emphasize that in spite of the existence of IM in normal samples, there are many more IM CpG sites in cancer (Fig. 1a and b in [18]). The enrichment of these types of CpG sites is a result of sample mixing. In iDMC selection, we attempt to pick CpG sites that contain information for cancer purity. Even though there might be some iDMCs that are results of IM and methylation heterogeneity, a majority of them are results of cancer-normal mixing, evidenced by the strong bimodality in the histogram of the beta values for iDMCs (Fig. 1e in [18]). Thus, we are able to estimate purity with good accuracy from the mode of the distribution. In spite of that, we want to point out that the level of heterogeneity depends on cancer type. Cancers with higher heterogeneity levels could have more “wrong” iDMCs selected and thus have biased purity estimation. In real data application, we recommend trying different numbers of iDMCs and examining the consistence in purity estimation.

It is important to note that in our purity estimation procedure, combining normal samples from different tissue types might increase the variation within the normal group and it was the reason why in the earlier version of InfiniumPurify we used matched controls in each cancer type to identify iDMCs. However, through comprehensive data analysis we notice that combining normal samples in fact produces comparable results. We believe this is a better approach and will have a wider application, for example, purity estimation can be performed for cancer types not included in TCGA. For any cancer type, as long as the sample size is reasonably large (e.g. ≥20), the iDMCs can be reliably detected by comparing the cancer to the universal normal controls and the purity can be estimated. We also want to point out that our DM calling (especially control-free DM calling) methods require relatively larger samples size compared with minfi, limma, or related tools. Thus, we envision that InfiniumPurify will be mostly applied to population level studies. Control-free DM calling also requires that the purities among samples are dispersed enough so that the statistical test can be reliably performed. To this end, we want to emphasize that the control-free DM calling method should be used with caution and normal controls should be profiled whenever possible.

Beyond differential methylation, analysis of other types of genomics data analysis such as differential expression between cancer and normal also suffer the complication of tumor purity. We believe similar principals proposed in this work can be applied to analyze the gene expression data, even though the detailed data modeling is different. This is also a research area we will explore in the near future.

## Conclusion

Tumor purity is an important factor of clinical tumor tissues reflecting both intrinsic property of a cancer type and how accurate these samples are collected. It could have a great impact on many cancer data analyses including differential expression, copy number alteration, differential methylation, genome-wide association studies, and EWAS. It is important to be able to estimate and adjust for tumor purities in these analyses. In this work, we develop a series of statistical methods for DNA methylation microarray data analysis in cancer, including purity estimation and DM calling with and without normal controls. The newly designed purity estimation procedure has greatly enhanced the application of InfiniumPurify for many cancer types with few or no normal samples. We estimate tumor purities of all tumor samples with 450 k data and show that our estimated purities are highly consistent with those from other popular tools. With consideration of purity, the DM calling results from cancer-normal comparison are shown to be more sensitive, accurate, and biologically meaningful. The control-free DM calling method provides a solution for data without normal control and provides new biological insights for more cancer types.

## Methods

### Purity estimation algorithm

The purity estimation algorithm by InfiniumPurify is illustrated in the purity estimation module in Fig. 1. For a given cancer type, we first collect all tumor samples and a set of normal samples to detect the informative differentially methylated CpG sites (iDMCs) and use those for purity estimation. A previous version of InfiniumPurify simply collects available normal samples of the corresponding cancer types to get iDMCs. However, for most cancer types in TCGA, there are not enough (or no) normal samples to obtain reliable iDMCs. In this updated version, we create a panel of the normal samples by taking two normal samples for each cancer type (one if there is only one normal sample for a cancer type). In total, we obtain 43 normal samples from 22 cancer types having normal samples and use them as the universal normal set for all cancer types (Additional file 3: Table S2). With these, we select DMCs between tumor and normal samples by rank-sum test and require that their variances of beta values are greater than 0.005 in tumor samples. Besides the rank-sum test, we also tried to use minfi in iDMC selection. The iDMCs selected from minfi are highly overlapped with those from rank-sum test and the estimated tumor purities are also highly correlated from procedures. In detail, the average overlap of top 1000 iDMCs by rank-sum test and minfi is 569 for all 32 cancer types (Additional file 3: Table S3) and the average purity correlation is over 0.9 (Additional file 2: Figure S17).

We then keep the top 1000 DMCs (based on *p* values from the rank-sum test) as iDMCs and used them for purity estimation (list of iDMCs are provided in Additional file 1: Material Section S4). Real data results show that the number of selected iDMCs has very little effect on the result. With this set of universal normal samples, iDMCs selection can be performed for data without normal controls. Moreover, the iDMCs selected for different cancer types based on TCGA data can be used for estimating purity for a single cancer dataset. In this case, users only need to provide a cancer type and the purity will be estimated based on the pre-determined iDMCs from TCGA data.

To estimate purity, iDMCs are first divided into hyper-methylated and hypo-methylated groups according to their mean beta values in tumor and normal samples. In detail, if an iDMC has higher mean methylation level tumor samples than normal, it will be assigned as a hyper-methylated group, and vice versa. Beta values of iDMCs in tumor samples are transformed according to the following procedure: hyper-methylated iDMCs remain unchanged and hypo-methylated iDMCs are changed to 1-beta values. Note that there is a small proportion of hyper-methylated iDMCs with beta values less than 0.5 and hypo-methylated iDMCs with beta values greater than 0.5. However, this transformation is performed regardless of the methylation level itself. We then apply density estimation with Gaussian kernel to the transformed methylation levels of the iDMCs. The mode of the density function is taken as the estimated purity. The estimated purities are then taken as known constants for downstream DM calling.

In the above procedure, iDMC selection is a key step for reliable purity estimation. However, iDMC selection could be affected by many factors including sample size, tumor stage, and heterogeneity. In particular, tumor heterogeneity is an intrinsic and commonly observed property of tumor samples and the level of heterogeneity depends on cancer type. Heterogeneous sites are more likely to be selected as iDMCs due to their high variance in tumor and difference between tumor and normal samples, which could bias the purity estimation. In this case, one can use different numbers of iDMCs in purity estimation and examine the stability of the results and then choose a proper number of iDMCs which gives the most stable result.

### Differential methylation analysis with normal control

The proposed DMC calling method works for data from one cancer type. The input raw data are beta values for *M* CpG sites from *n*_1_ cancer and *n*_0_ normal samples. We first transform the beta values using an arcsine transformation: *f*(*x*) = arcsin (2*x* − 1). The transformation is necessary because the transformed data follow Gaussian distribution much better compared to the raw data, especially at the boundaries (0 and 1). This allows us to use a linear model with Gaussian noise in following method. The arcsine is a “variance stabilization transformation” for random variables from a beta distribution. Such transformation possesses several advantages over more commonly used logistic (logit) transformation. First, it stabilizes variance, e.g. the variance does not depend on the mean anymore. This greatly reduces the heteroscedasticity problem in regression. Second, it is more linear than logit. The methylation level from mixed cancer-normal samples is assumed to be a weighted average of those from the pure samples and the signal mixing is at the original scale. Having a more linear transformation allows us to use a linear model for transformed data with better approximation. Previous work on differential methylation analysis from bisulfite sequencing uses the arcsine transformation and obtains good results [58].

For CpG site *i*, denote the transformed beta values from normal samples as *X*_*i*_, and assume *X*_*i*_ ∼ *N*(*m*_*i*_, *σ*_*i*_^2^). Denote the transformed beta values from “pure” cancer samples as *Y*_*i*_ and assume *Y*_*i*_ = *X*_*i*_ + *δ*_*i*_. *δ*_*i*_ is a random variable representing the difference between cancer and normal samples. It is assumed that *δ*_*i*_ ∼ *N*(*μ*_*i*_, *τ*_*i*_^2^). With *X*_*i*_ and *Y*_*i*_ from a number of samples, the differential methylation detection is achieved by hypothesis testing: *H*_0_ : *μ*_*i*_ = 0. However, in practice, the data from pure cancer sample *Y*_*i*_ is not observed. Instead, we observed the signal from mixed cancer-normal samples, denoted by *Y*_*i*_ '. For cancer sample *s* with known purity *λ*_*s*_, we have *Y*_*is*_ ' = (1 − *λ*_*s*_)*X*_*is*_ + *λ*_*s*_ *Y*_*is*_ = (1 − *λ*_*s*_)*X*_*is*_ + *λ*_*s*_(*X*_*is*_ + *δ*_*is*_) = *X*_*is*_ + *λ*_*s*_ *δ*_*is*_, so *Y*_*is*_ ' ∼ *N*(*m*_*i*_ + *λ*_*s*_ *μ*_*i*_, *σ*_*i*_^'2^). Here, *σ*_*i*_'^2^ is the variance for *Y*_*is*_' , and *σ*_*i*_'^2^ ≠ *σ*_*i*_^2^. It is worth mentioning that *X*_*i*_ and *δ*_*i*_ have moderate negative correlation in real data (Additional file 2: Figure S18). This is expected because lowly methylated CpG sites in normal samples tend to be hyper-methylated and highly methylated CpG sites in normal samples tend to be hypo-methylated. The negative correlation, however, does not affect the general design of our model. This derivation shows that because of the presence of *λ*_*s*_, directly testing the mean differences between *X*_*is*_ and *Y*_*is*_ *'* is not equivalent to testing *H*_0_ : *μ*_*i*_ = 0. This also shows that the tumor purity has multiplicative effect on differential methylation (the same applies to different expression), instead of additive. Therefore, the existing model for differential methylation or differential expression with the consideration of tumor purity by using purity as an additive covariate [46] is incorrect statistically. We designed the following method based on a simple linear model and the generalized least square procedure to take *X*_*is*_ and *Y*_*is*_ *'* as input data and test *μ*_*i*_ = 0.

For CpG site *i*, we denote all input data by a vector ${Z}_i={\left[{X}_{i1},{X}_{i2},\dots, {X}_{i{ n}_0},{Y}_{i1}\prime, {Y}_{i2}\prime, \dots, {Y}_{i{ n}_1}\prime \right]}^T$. The first *n*_0_ items are numbers from normal samples and the next *n*_1_ items are from cancer samples. The input data can be represented by following the linear model: *Z*_*is*_ = *m*_*i*_ + *a*_*s*_ *μ*_*i*_ + *ϵ*_*s*_, *s* = 1, 2, …, *n*_0_ + *n*_1_, where *a*_*s*_ = 0 when *s* ≤ *n*_0_ and *a*_*s*_ = *λ*_*s*_ when *n*_0_ < *s* ≤ *n*_1_. In this model, *μ*_*i*_ is the parameter of interest that will be tested. The residual variances are *σ*_*i*_^2^ and *σ*_*i*_'^2^, respectively, for normal and cancer groups. This method essentially uses tumor purity as an experimental design factor in a linear model, so that the correct inference on differential methylation can be obtained.

The parameter estimation can be performed by following the generalized least square method. For a CpG site, let

$$ Z=\left[\begin{array}{l}{X}_1\\ {}{X}_2\\ {}\vdots \\ {}{X}_{n_0}\\ {}{Y}_1\hbox{'}\\ {}{Y}_2\hbox{'}\\ {}\vdots \\ {}{Y}_{n_1}\hbox{'}\end{array}\right], W=\left[\begin{array}{cc}\hfill \begin{array}{c}\hfill 1\hfill \\ {}\hfill \begin{array}{c}\hfill 1\hfill \\ {}\hfill \vdots \hfill \end{array}\hfill \\ {}\hfill 1\hfill \end{array}\hfill & \hfill \begin{array}{c}\hfill 0\hfill \\ {}\hfill \begin{array}{c}\hfill 0\hfill \\ {}\hfill \vdots \hfill \end{array}\hfill \\ {}\hfill 0\hfill \end{array}\hfill \\ {}\hfill \begin{array}{c}\hfill 1\hfill \\ {}\hfill \begin{array}{c}\hfill 1\hfill \\ {}\hfill \vdots \hfill \end{array}\hfill \\ {}\hfill 1\hfill \end{array}\hfill & \hfill \begin{array}{c}\hfill {\lambda}_1\hfill \\ {}\hfill {\lambda}_2\hfill \\ {}\hfill \begin{array}{c}\hfill \vdots \hfill \\ {}\hfill {\lambda}_{n_1}\hfill \end{array}\hfill \end{array}\hfill \end{array}\right],\beta =\left[\begin{array}{c}\hfill m\hfill \\ {}\hfill \mu \hfill \end{array}\right],\mathrm{and}\;\epsilon =\left[\begin{array}{l}{\epsilon}_1\\ {}{\epsilon}_2\\ {}\vdots \\ {}{\epsilon}_{n_0}\\ {}{\epsilon}_{n_0+1}\\ {}{\epsilon}_{n_0+2}\\ {}\vdots \\ {}{\epsilon}_{n_0+{n}_1}\end{array}\right], $$

where *Z* is a vector for transformed methylation levels in *n*_0_ normal and *n*_1_ tumor samples, *W* is a *n*_0_ + *n*_1_ by 2 design matrix with *n*_0_ 0’s and tumor purities in the second column, *β* is the linear model parameter to be determined, and *ϵ* is the error term. The linear regression model can be formulized as *Z* = *Wβ* + *ϵ*, and the model parameters can be solved by the following normal equation,

$$ \widehat{\beta}={\left({W}^T W\right)}^{-1}{W}^T Z\triangleq H Z $$, where *H* = (*W* ^*T*^ *W*)^−1^ *W* ^*T*^, and $$ v a r\left(\widehat{\beta}\right)= Hvar(Z){H}^T $$.

*var*(*Z*) is in the form of $$ \left[\begin{array}{cc}\sum & 0\\ {}0& \sum \end{array}^{\prime}\right] $$, where $$ \sum ={\left[\begin{array}{ccc}{\sigma}^2& 0& 0\\ {}0& \ddots & 0\\ {}0& 0& {\sigma}^2\end{array}\right]}_{n_0\times {n}_0} $$, $$ \sum^{\prime }={\left[\begin{array}{c}\sigma {\prime}^2\kern0.24em 0\kern0.48em 0\\ {}0\kern0.48em \ddots \kern0.36em 0\\ {}0\kern0.48em 0\kern0.48em \sigma {\prime}^2\end{array}\right]}_{n_1\times {n}_1} $$.

So $$ var\left(\widehat{\beta}\right)= Hvar(Z){H}^T=\left[\begin{array}{cc}{H}_1& {H}_2\end{array}\right]\;\left[\begin{array}{cc}\sum & 0\\ {}0& \sum^{\prime}\end{array}\right]\left[\begin{array}{c}{H}_1^T\\ {}{H}_2^T\end{array}\right]={H}_1\sum {H}_1^T+{H}_2\sum^{\prime }{H}_2^T $$, and $v a r\left(\widehat{\beta}\right)$ can be obtained with *σ* ^2^ and *σ'* ^2^, the residual variances from normal and cancer groups. To estimate *σ* ^2^ and *σ'* ^2^, once we have $\widehat{\beta}$, regression residuals are $\widehat{\in}= Z- W\widehat{\beta}$, then,

$$ {\sigma}^2=\frac{{\displaystyle {\sum}_{i=1}^{n_0}}{\widehat{\mathit{\in}}}_i^2}{n_0-2},\kern0.36em {\upsigma \mathrm{\hbox{'}}}^2=\frac{{\displaystyle {\sum}_{i={n}_0+1}^{n_0+{n}_1}}{\widehat{\mathit{\in}}}_i^2}{n_1-2}. $$

We applied a shrinkage estimator, similar to the one proposed in [59], on the estimated cancer/normal variances and obtained ${\tilde{\sigma}}^2$ and ${{\overset{\sim }{\sigma}}^{\prime}}^2$. The procedure shrinks all residual variances to the geometric mean and stabilizes the estimates.

After getting $\widehat{\beta}$ and $v a r\left(\widehat{\beta}\right)$, the Wald test statistics for testing *H*_0_ : *μ* = 0 is

$$ t=\frac{{\widehat{\beta}}_{\left[2\right]}\ }{{\sqrt{\  var\left(\widehat{\beta}\right)}}_{\left[2,2\right]}}, $$

where ${\widehat{\beta}}_{\left[2\right]}$ is the second item of $\widehat{\beta}$, ${\sqrt{var\left(\widehat{\beta}\right)}}_{\left[2,2\right]}$ is the [2, 2] element of the matrix $\sqrt{\  var\left(\widehat{\beta}\right)}$.

Finally, we assume the Wald test follow a *t* distribution with *n*_0_ + *n*_1_ − 2 degrees of freedom to obtain nominal *p* values. Adjustment of multiple testing can be done by applying canonical procedure to compute FDRs [60].

### Control-free differential methylation detection

Following the notations in the cancer-normal DM calling method, we have *Y*_*is*_ ' ∼ *N*(*m*_*i*_ + *λ*_*s*_ *μ*_*i*_, *σ*_*i*_'^2^) and wish to test the difference in average methylation levels between cancer and normal, e.g. *μ*_*i*_ = 0, without control data. With known tumor purities *λ*_*s*_, the hypothesis testing can be performed even without control data. Through a simple linear regression using the data from tumor samples (*Y*_*is*_ ') as response and tumor purities (*λ*_*s*_) as independent variables, the difference in means (*μ*_*i*_) is the slope in the regression and can be tested. We want to note that the test statistics from such regression is equivalent to the Pearson’s correlation between *Y*_*is*_ ' and *λ*_*s*_, but the regression procedure offers some flexibility to incorporate other criteria. We find that sometimes a CpG site has large test statistics but relatively small effect size, due to small standard error. To limit such effect, we use the posterior probability Pr(|*μ*_*i*_| > *c*) to rank the CpG sites, where *c* is a user defined quantity to require the difference between cancer and normal to be larger than a threshold. Using the test statistics (or correlation between *Y*_*is*_ ' and *λ*_*s*_ is equivalent to setting *c =* 0). In practice, we used *c* = 0.1 and found that it provides better performance than using *c* = 0.

## Additional files

Additional file 1: Materials **S1.** Genomic locations of iDMCs. **S2.** Simulation. **S3.** Control-free DM calling by using universal normal samples. (PDF 3492 kb)
Additional file 2: Figures S1 – S18. (PDF 10334 kb)
Additional file 3: Tables S1 – S4. (PDF 75 kb)

## Abbreviations

DMCs: differentially methylated CpG sites
DMRs: differentially methylated regions
ICGC: The International Cancer Genome Consortium
iDMCs: informative differentially methylated CpG sites
RRBS: reduced representation bisulfite sequencing
TCGA: The Cancer Genome Atlas
WGBS: whole genome bisulfite sequencing
