# Supplementary material for: Estimating and accounting for tumor purity in the analysis of DNA methylation data from cancer studies
Source: Genome Biol. 2017 Jan 25;18:17. doi: 10.1186/s13059-016-1143-5 (PMC5267453; doi:10.1186/s13059-016-1143-5)
Supplement: Additional file 1: — Materials S1. Genomic locations of iDMCs. S2. Simulation. S3. Control-free DM calling by using universal normal samples. (PDF 3492 kb) [file 13059_2016_1143_MOESM1_ESM.pdf]

S1. Genomic locations of iDMCs

We carefully explore the genomic locations of the iDMCs from different cancer types.

Averaging across all cancer types, 22% of the iDMCs are located at the transcriptional start site (TSS), 3% are at transcriptional end site (TES), 11% at exonic regions, 32% at intronic regions, and 31% at intergenic regions. Compared with the location of all CpG sites on the 450k arrays, the iDMCs are significantly depleted at the TSS (p=6.2e-05), and enriched at the intergenic regions (p= 0.00012). A comparison of the iDMC locations is shown in the figure below. These results demonstrate that the iDMCs are more likely to appear at relatively less important regions in the genome.

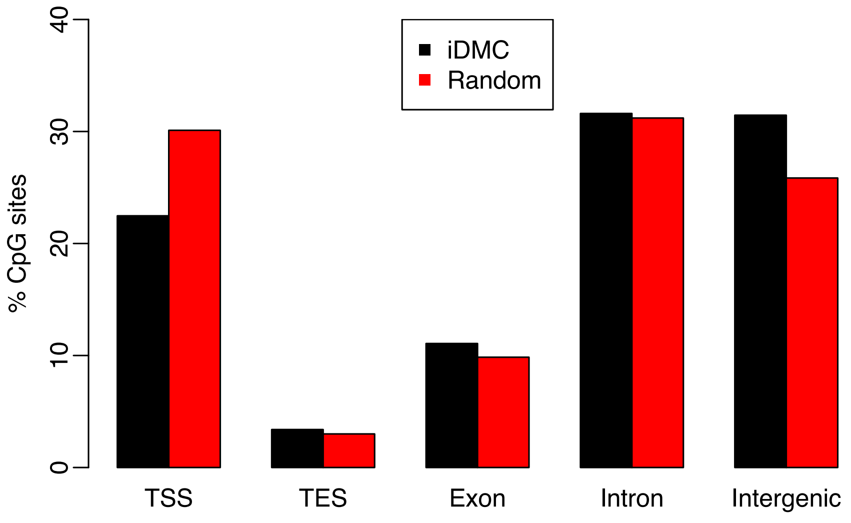

We further explore the genes to which the iDMCs are close. We associate each iDMC to a gene if it's located within 3000 bps to the gene. On average, the top 1000 iDMCs are located in 432 genes (number of genes containing iDMC from different cancer types is shown in the figure below), Most (89%) of the iDMC-bearing genes contain only 1 or 2 iDMCs, thus the locations of iDMCs are rather dispersed. The spatial diversity is desirable and potentially more robust, because the purity estimation result won't be overly influenced by the differential methylation by a few genes.

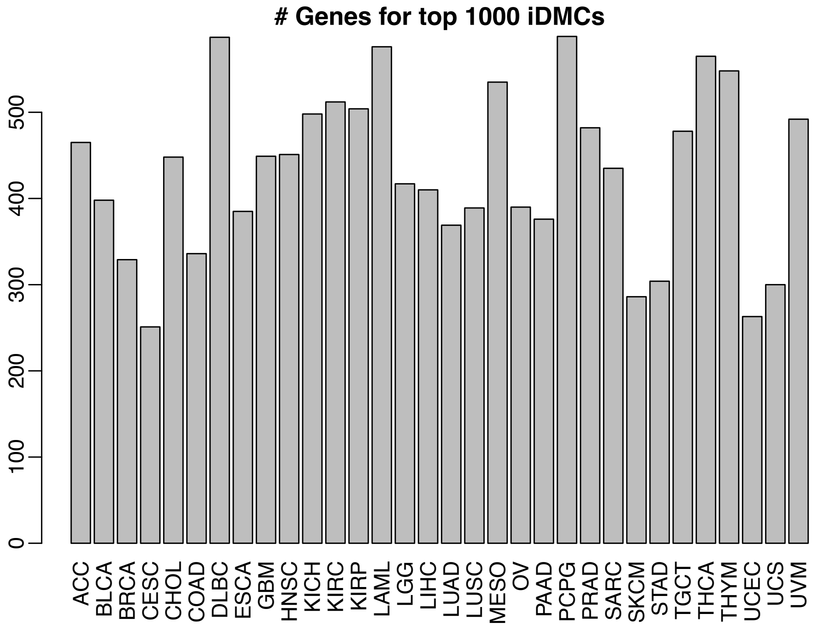

Next, we explore the overlap of iDMCs from different cancer types. Overall, the overlaps are rather low: the average pairwise overlaps is only 2.8%. At the gene level, average overlap of iDMC-bearing genes among different cancer types is 3.2%, still very low. These results demonstrate the cancer type specificity of iDMCs, thus it is necessary to obtain a set of iDMCs for each cancer.

S2. Simulation

In order to guarantee that the simulated data have characteristics matching the real data, we used LUAD data as template in our simulation. Under the assumption that the beta values for each CpG site follow a beta distribution, we estimated the distributional parameters (α and β) for all CpG sites from the LUAD data. We then simulated normal and pure cancer beta values from these beta distributions. The beta values for tumor samples were generated by mixing the simulated pure normal and tumor data with randomly generated tumor purities.

We applied different methods, including minfi, RefFreeEWAS, and InfiniumPurify on the simulated data to call DMC, and compared their performances by the ROC curves. Because the true mean methylation

levels are known, we can construct the gold standards for comparison. The DM statuses for all CpG sites are defined as following. For a CpG site, if the absolute difference (defined as Δ) of the true methylation levels between normal and pure cancer samples is less than 0.05, it is deemed as non-DM. If the absolute difference is greater than a threshold, it is defined as DM. We varied the thresholds to define DM, so that the performances of DM calling can be compared under different signal to noise ratios (SNRs). We also varied the sample sizes by using 10, 50 and 100 samples in each group. The ROCs curves from the simulations are shown in the Figure below.

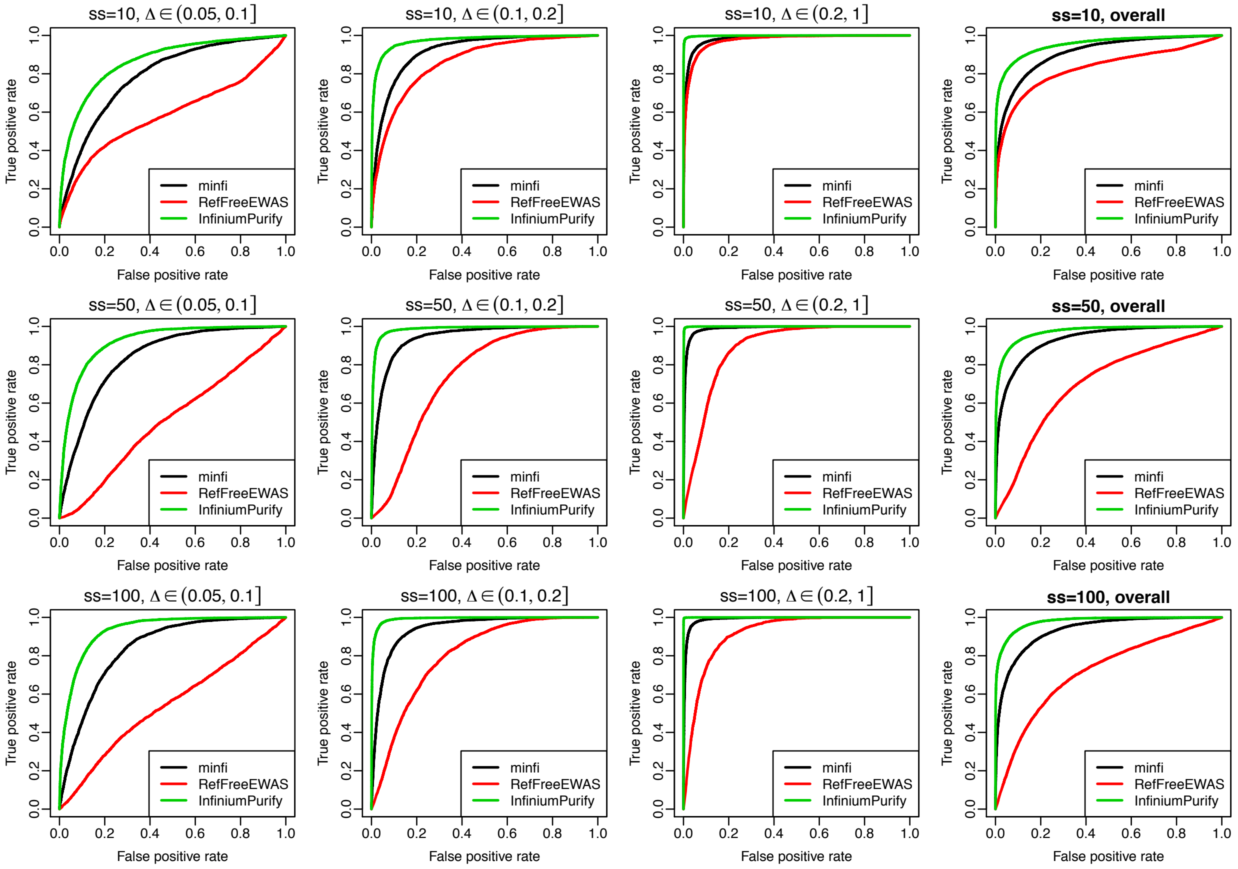

Under all simulation scenarios, InfiniumPurify provides the best results, followed by minif. RefFreeEWAS doesn't perform well, because it is not designed to for this type of comparison, as discussed in the paper. It is also clear that when SNR is larger, all methods perform better. For example, when DM is defined as absolute difference in mean methylation is greater than 0.2, both minfi and InfiniumPurify have close to

perfect performance. This is because the greater effect size can mostly out-weigh the noises brought by the purity. Results also show that greater sample size improves the performances for both minfi and InfiniumPurify. Overall, these real data based simulation results demonstrate the robustness and accuracy of InfiniumPurify in handling the DM calling in cancer study when tumor purity is a concern.

S3. Control-free DM calling by using universal normal samples

We also tried another possible DM calling solution when control data is unavailable: to use a universal set of normal samples as controls. We compared the results from using universal normal and the proposed control-free DM calling. The results from using matched normal are used as gold standard. We constructed ROC curves and computed their area under curve (AUCs) for a number of cancer types. The AUC values are listed in the table below.

Tumor type	Control free	Universal Normal
BLCA	0.947473765	0.959414333
BRCA	0.904318612	0.865625316
COAD	0.908875616	0.888440283
HNSC	0.884496132	0.911573403
KIRC	0.748469555	0.658095217
KIRP	0.755698252	0.767718742
LIHC	0.899391133	0.882225686
LUAD	0.915178856	0.909303391
LUSC	0.835539211	0.854412187
PRAD	0.903448031	0.854861961
THCA	0.873077179	0.811470438
UCEC	0.903636463	0.904246968

Overall, we found that DMCs detected from control free method is slightly better than using universal normal (average AUC is 0.8733 for control free method, and 0.8556 for using universal normal). In some cases, control free method show rather significant improvement for cancer types with low AUC, including

KIRC and THCA. These results demonstrate that the control-free DM calling method serves at least as a nice alternative to using universal normal, and the results can be better in some situation.

S4. P and q-values of iDMCs for different tumor types.

We list the p/q-values, genomic location and associated genes of selected iDMCs for different tumor types as follows:

https://bitbucket.org/zhengxiaoqi/infiniumpurify/raw/b0e0a0b08c43410e8194352aeb1da1cd3d733da0/iDMC.zip
