# Supplementary material for: Estimating and accounting for tumor purity in the analysis of DNA methylation data from cancer studies
Source: Genome Biol. 2017 Jan 25;18:17. doi: 10.1186/s13059-016-1143-5 (PMC5267453; doi:10.1186/s13059-016-1143-5)

Supplemental Figures for "Estimating and accounting for tumor purity in the analysis of DNA methylation data from cancer studies"

Xiaoqi Zheng[1,$,*], Naiqian Zhang[2,$], Hua-Jun Wu[3] and Hao Wu[4,*]

[1] Department of Mathematics, Shanghai Normal University. Shanghai 200234, China

[2] Department of Mathematics, Weifang University. Weifang, Shandong 261061, China

[3] Department of Biostatistics and Computational Biology, Dana-Farber Cancer Institute and Harvard School of Public Health, Boston 02215, MA, USA

[4] Department of Biostatistics and Bioinformatics, Rollins School of Public Health, Emory University. 1518 Clifton Road. Atlanta, Georgia 30322, USA

[$] These authors contributed equally to this work.

* To whom correspondence should be addressed. Tel: +1(404) 727-8633; Fax: (404)727-1370; Email: hao.wu@emory.edu (HW) or xqzheng@shnu.edu.cn (XZ)

Supplemental Figure 1. Comparison of tumor purities estimated by InfiniumPurify and ESTIMATE for each individual cancer type.

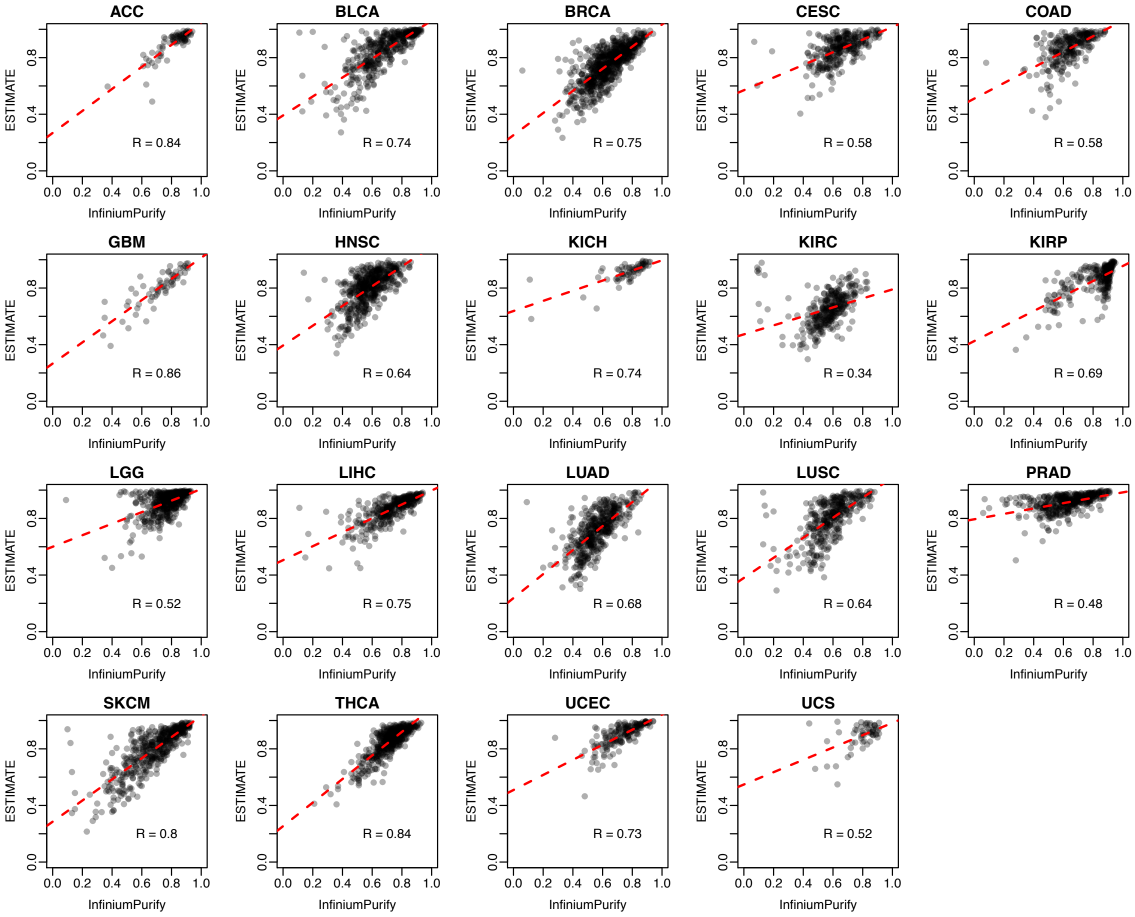

Supplemental Figure 2. Comparison of tumor purities estimated by InfiniumPurify and ABSOLUTE for each individual cancer type.

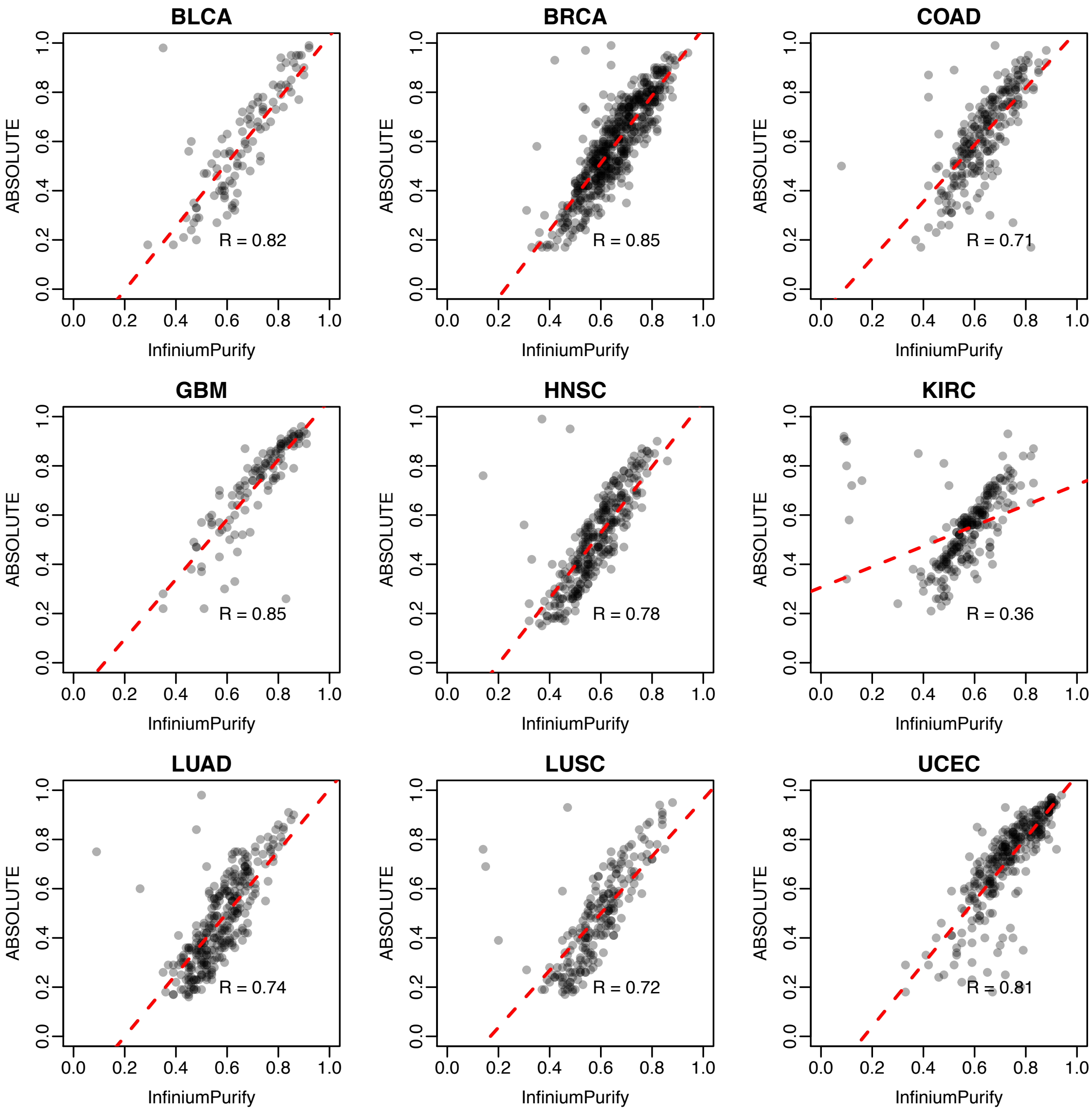

Supplemental Figure 3. Comparison of tumor purities estimated by InfiniumPurify and LUMP for each individual cancer type.

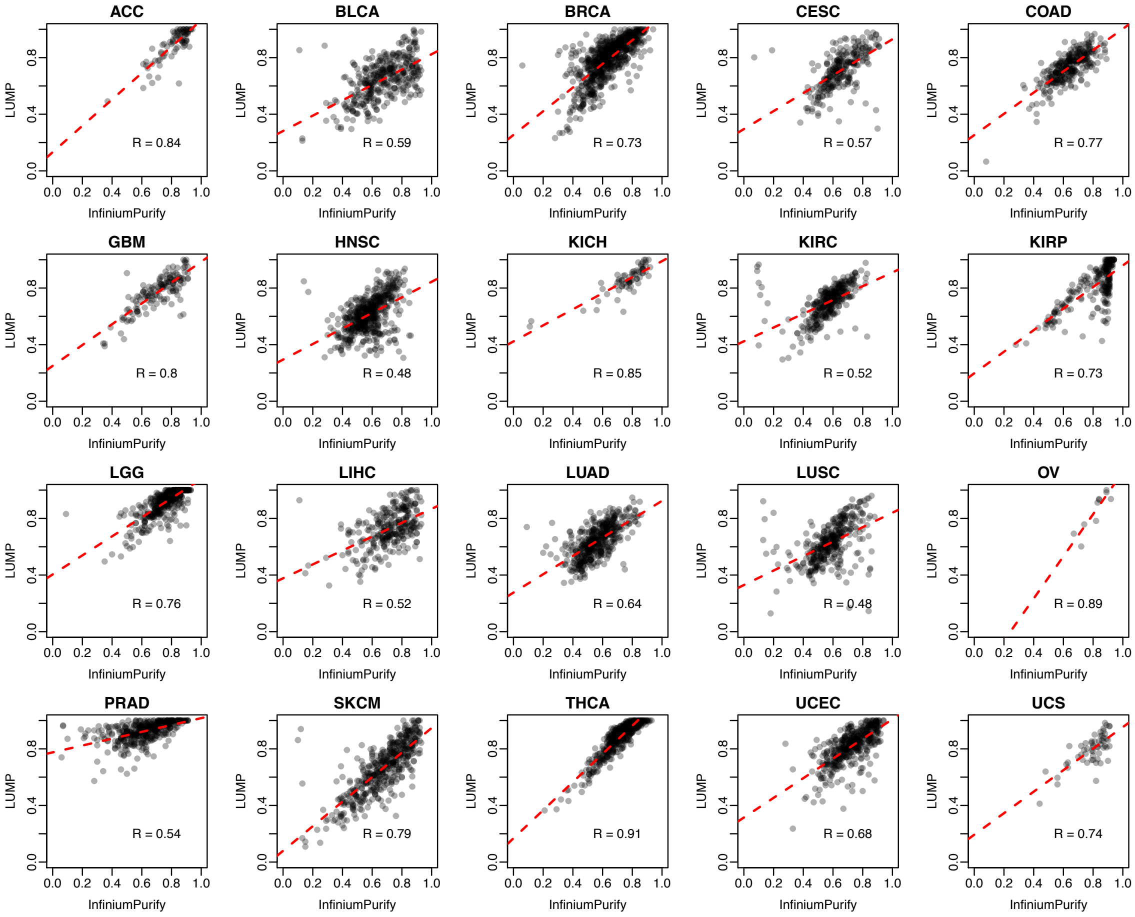

Supplemental Figure 4. Comparison of tumor purities estimated by InfiniumPurify and IHC for each individual cancer type.

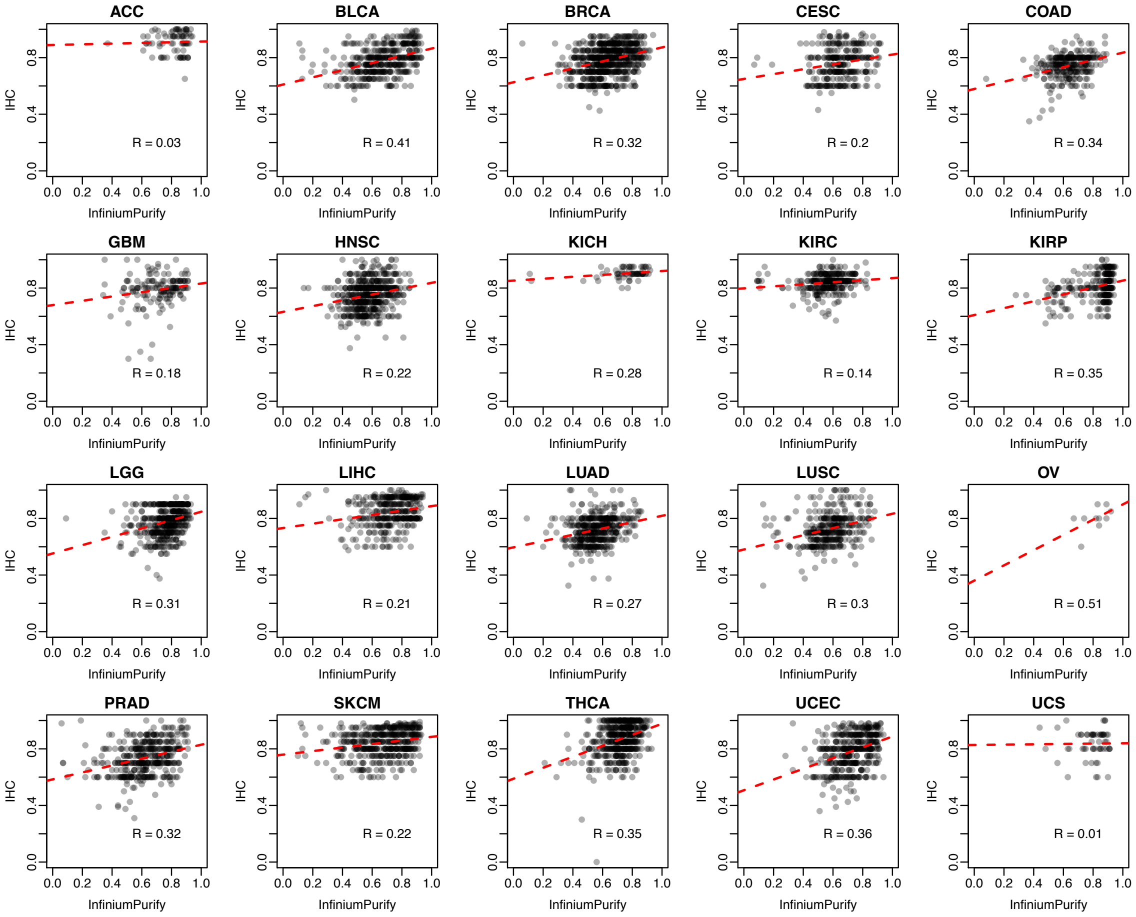

Supplemental Figure 5. Comparison of tumor purities estimated by InfiniumPurify and CPE for each individual cancer type.

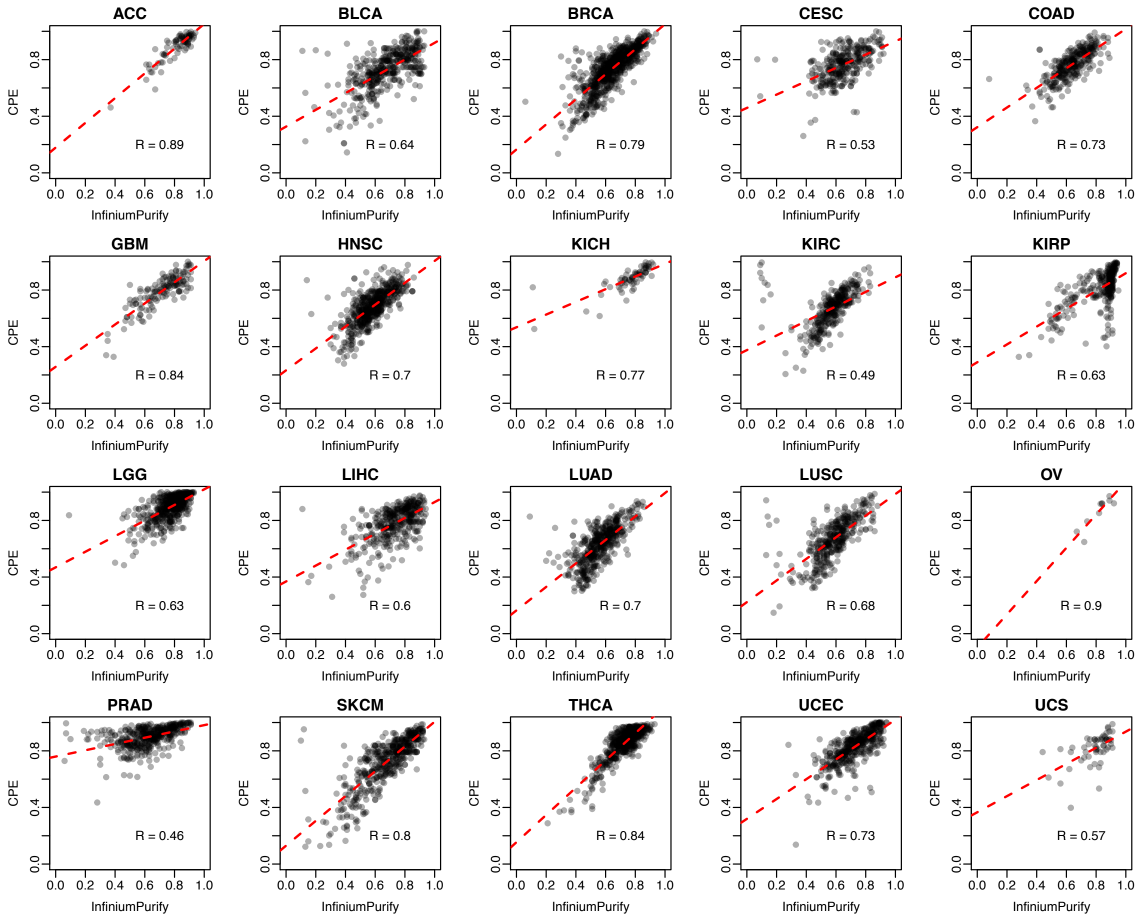

Supplemental Figure 6. Correlation of tumor purities by InfiniumPurify and other methods for different cancer types in TCGA.

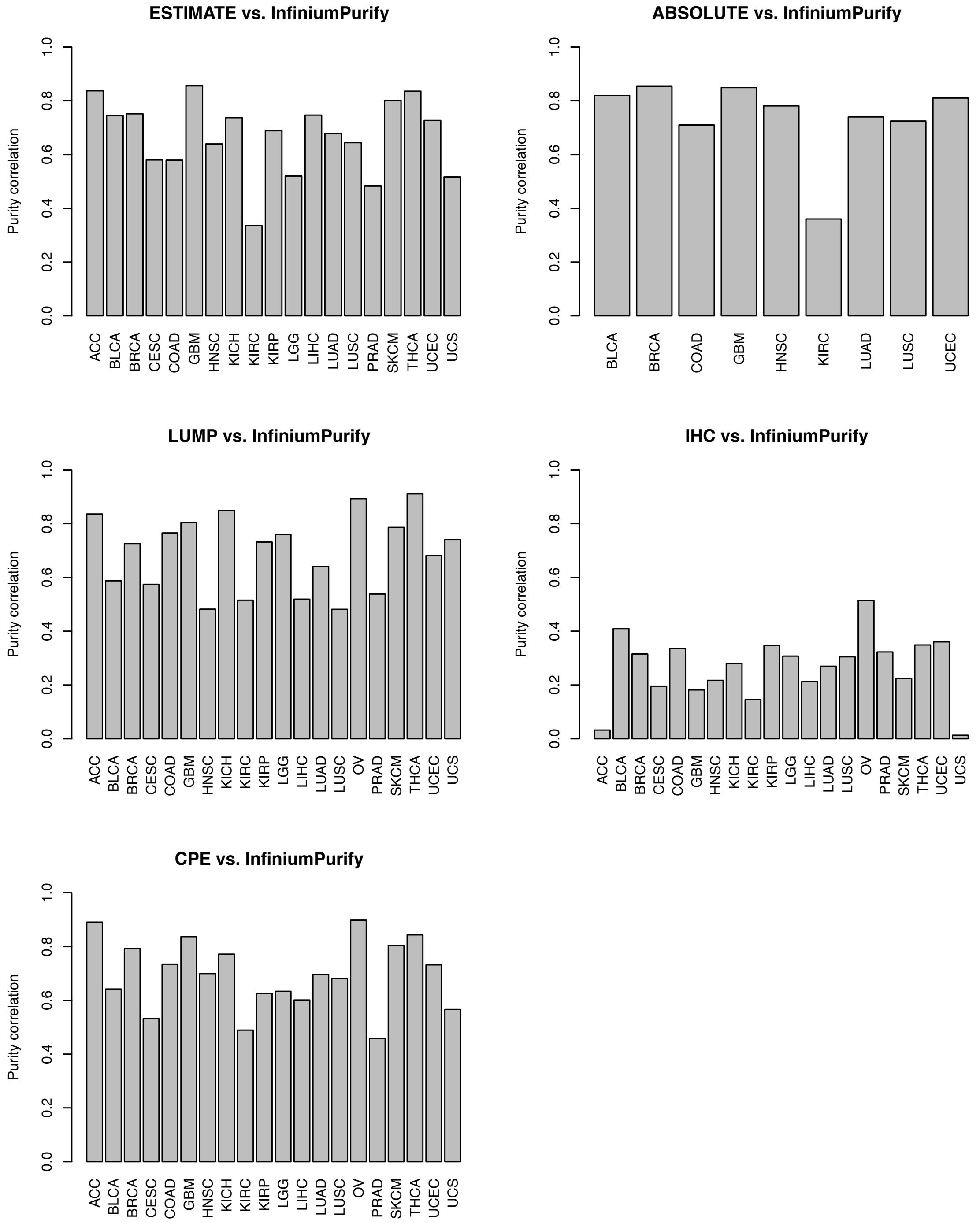

Supplemental Figure 7. Tumor purities estimated using blood controls compared with those using universal normal controls.

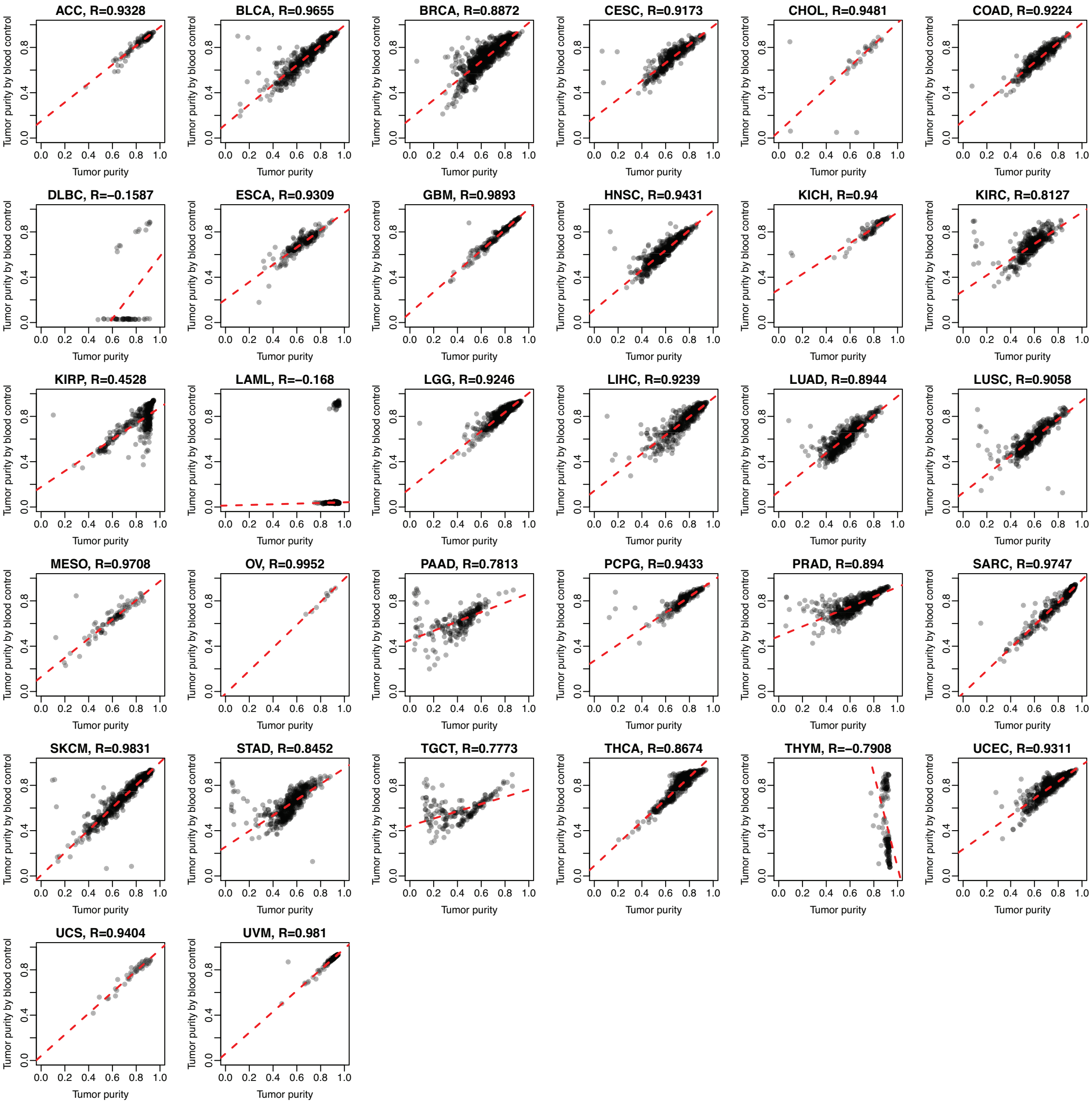

Supplemental Figure 8. Correlation between tumor purities estimated using blood controls with purities estimated by other available tools.

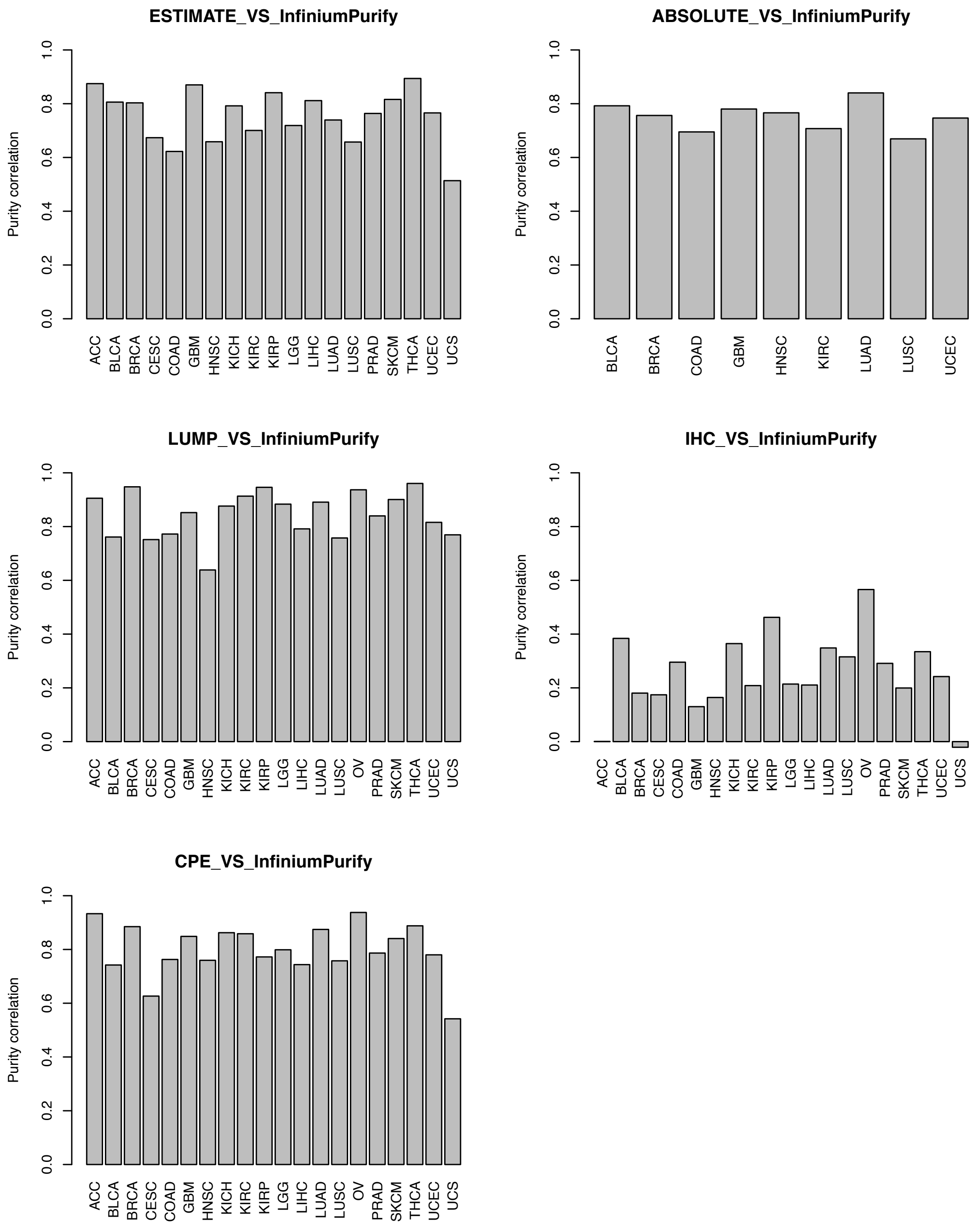

Supplemental Figure 9. Consistence between tumor purities estimated from 10-fold cross-validation and those by the whole data set.

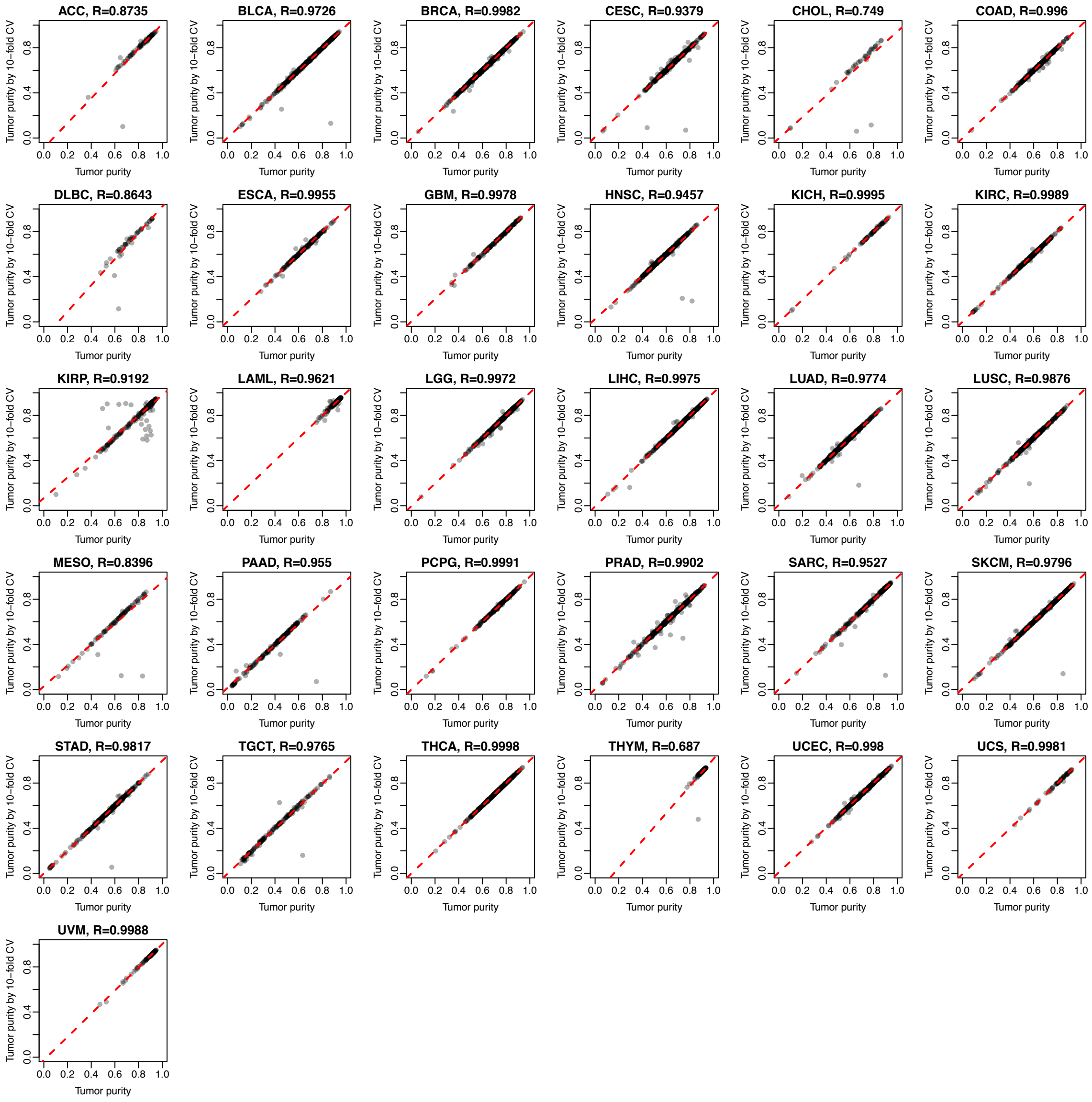

Supplemental Figure 10. Overlaps of DMCs called from different methods.

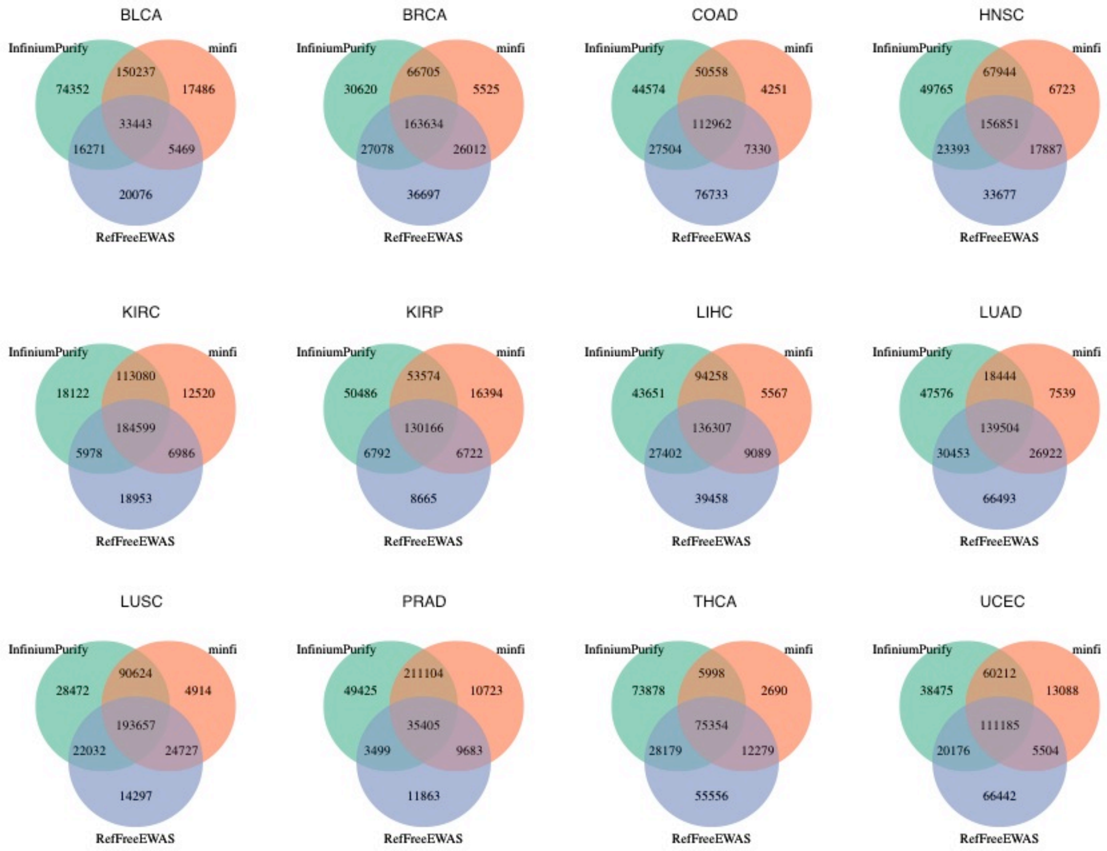

Supplemental Figure 11. Absolute methylation differences between matched tumor and normal samples for InfiniumPurify exclusive, minfi exclusive and common DMCs for BRCA samples.

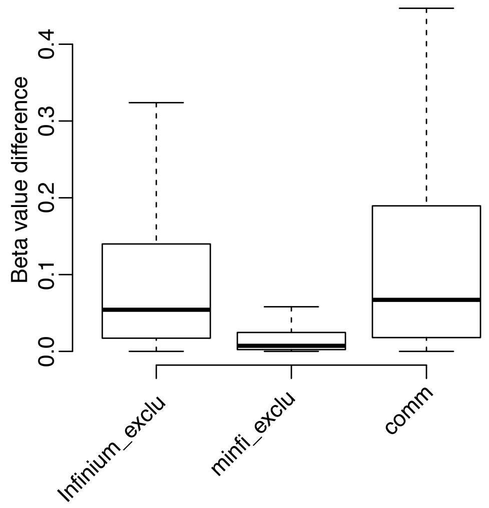

Supplemental Figure 12. Inter-cancer correlations of statistics by InfiniumPurify (a), minfi (b) and RefFreeEWAS (c).

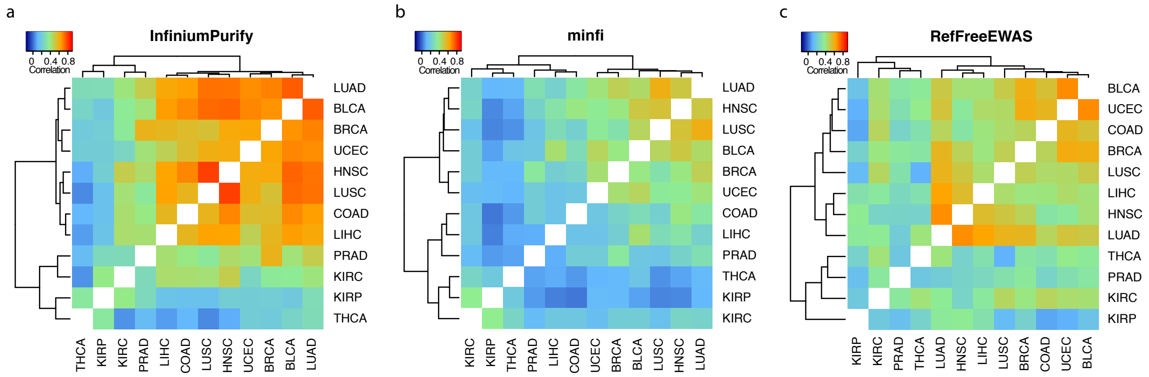

Supplemental Figure 13. Enrichment of top 1000 differentially methylated genes in KEGG pathways related to different cancers.

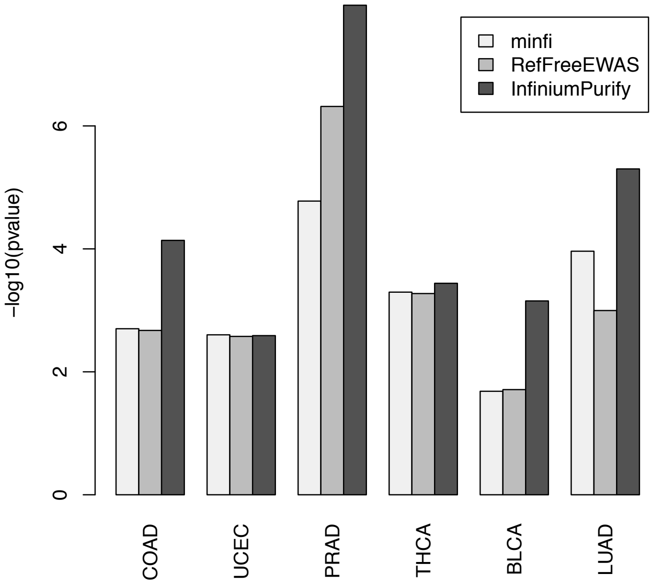

Supplemental Figure 14. Examples showing DMCs that are only detected by InfiniumPurify. Left panel shows their methylation level distributions in tumor and normal samples. Middle panel shows correlation between purities and methylation levels. Right panel shows methylation levels of normal and tumor samples after correcting for tumor purities.

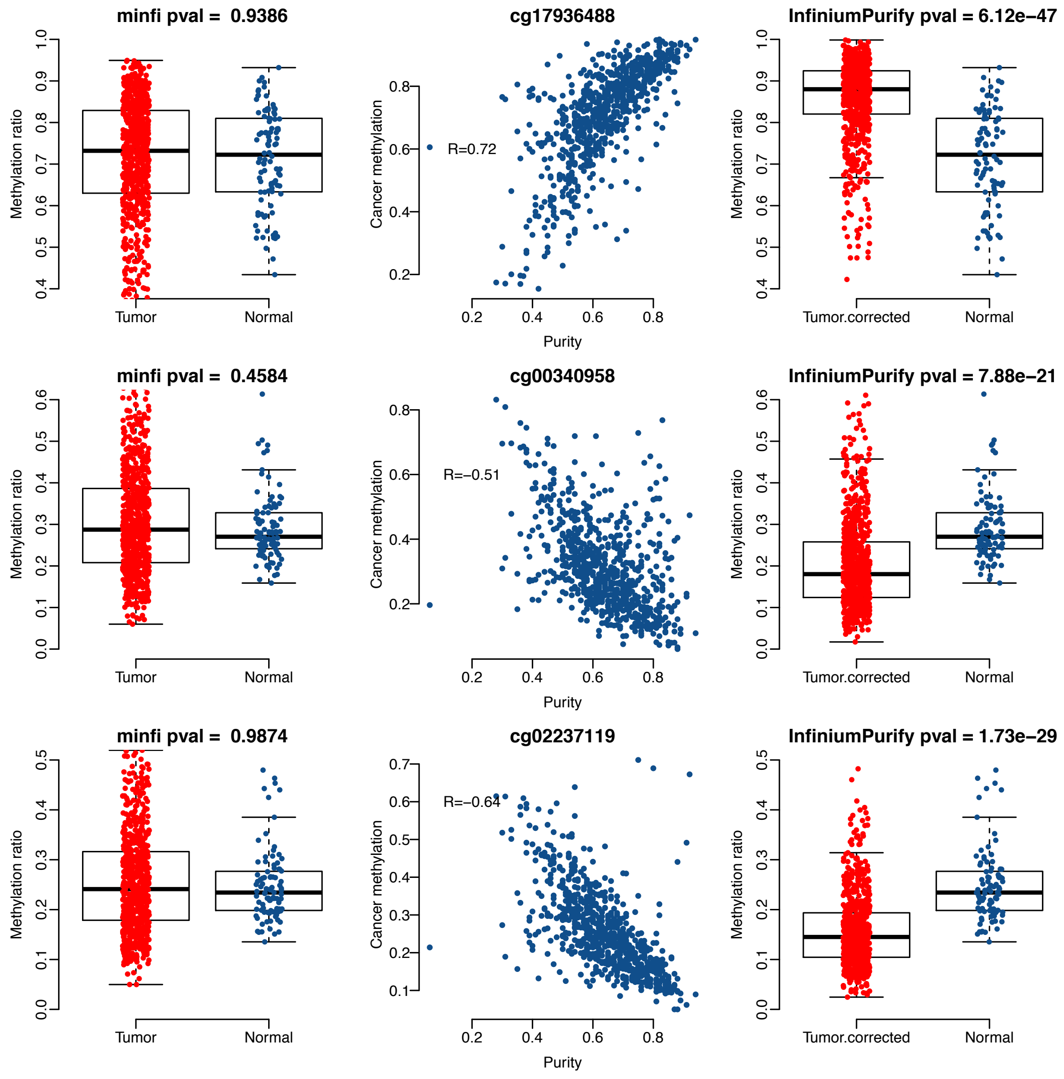

Supplemental Figure 15. ROC curves of control-free DMC calling for all TCGA cancer types with more than 20 normal samples.

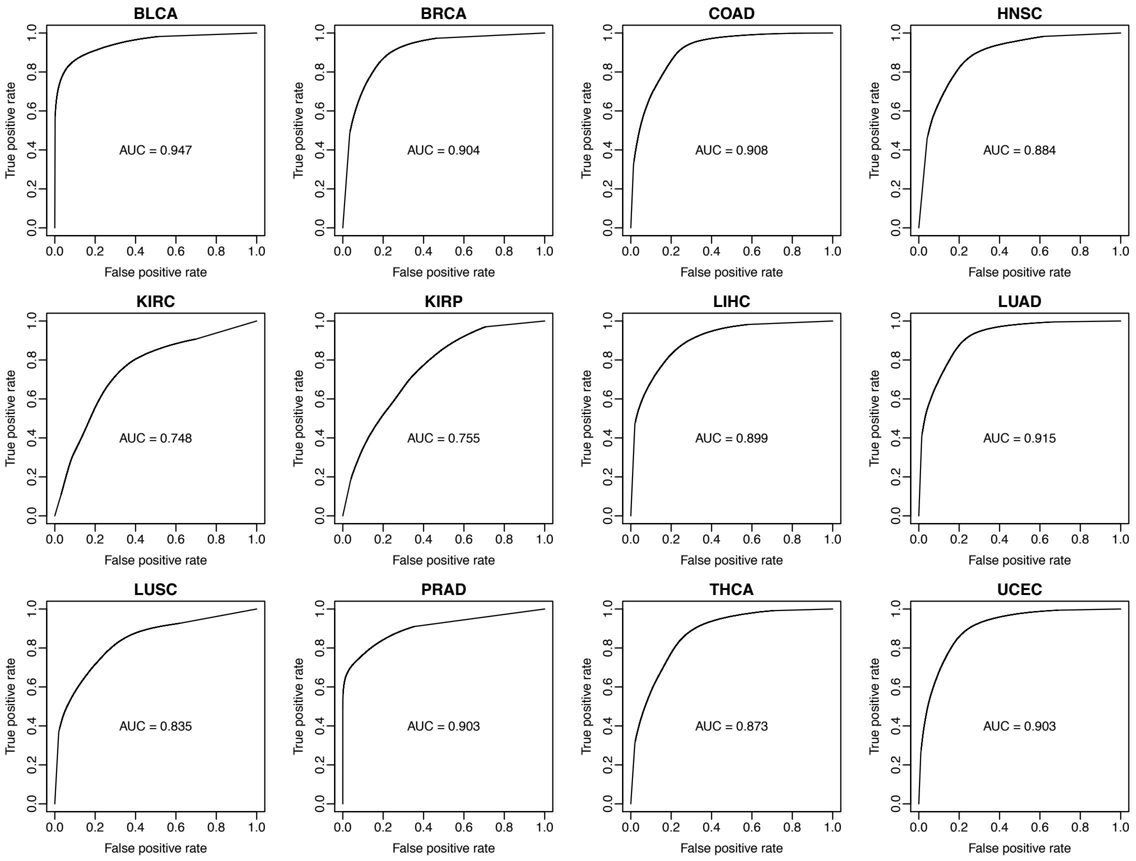

Supplemental Figure 16. TDR curves of control-free DMC calling for all qualified TCGA cancer types.

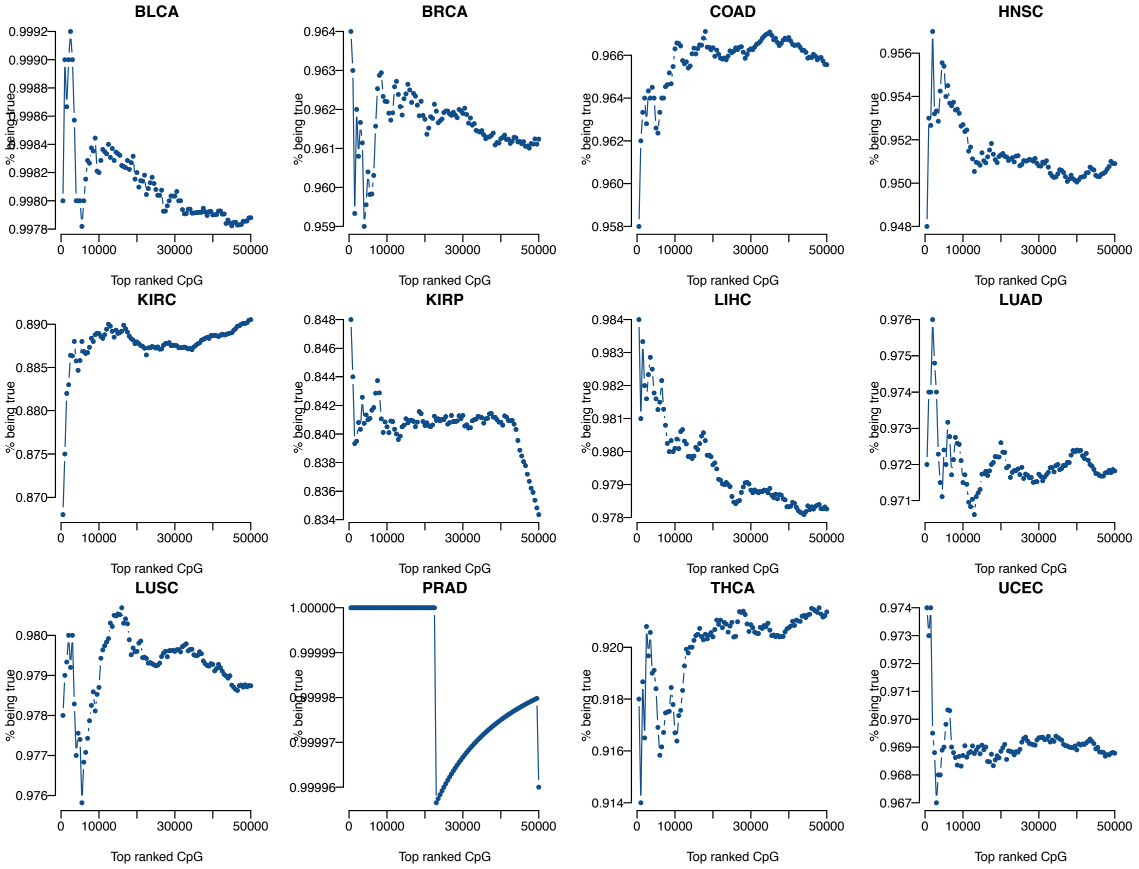

Supplemental Figure 17. Purity correlation estimated using Rank-sum test and minfi for iDMC selection.

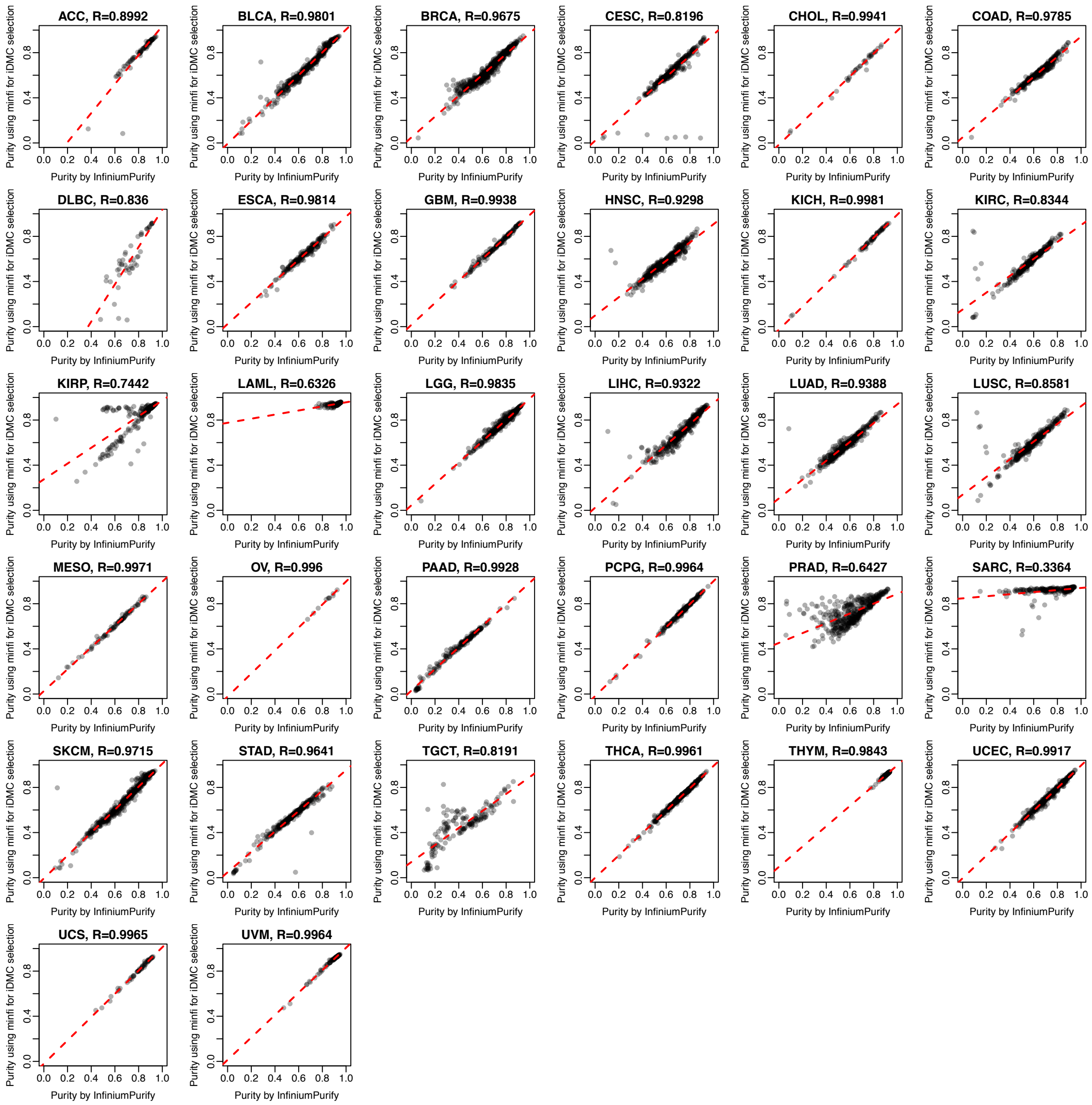

Supplemental Figure 18. Correlation between averaged methylation level of each CpG site in normal samples and their methylation difference between tumor and normal samples.

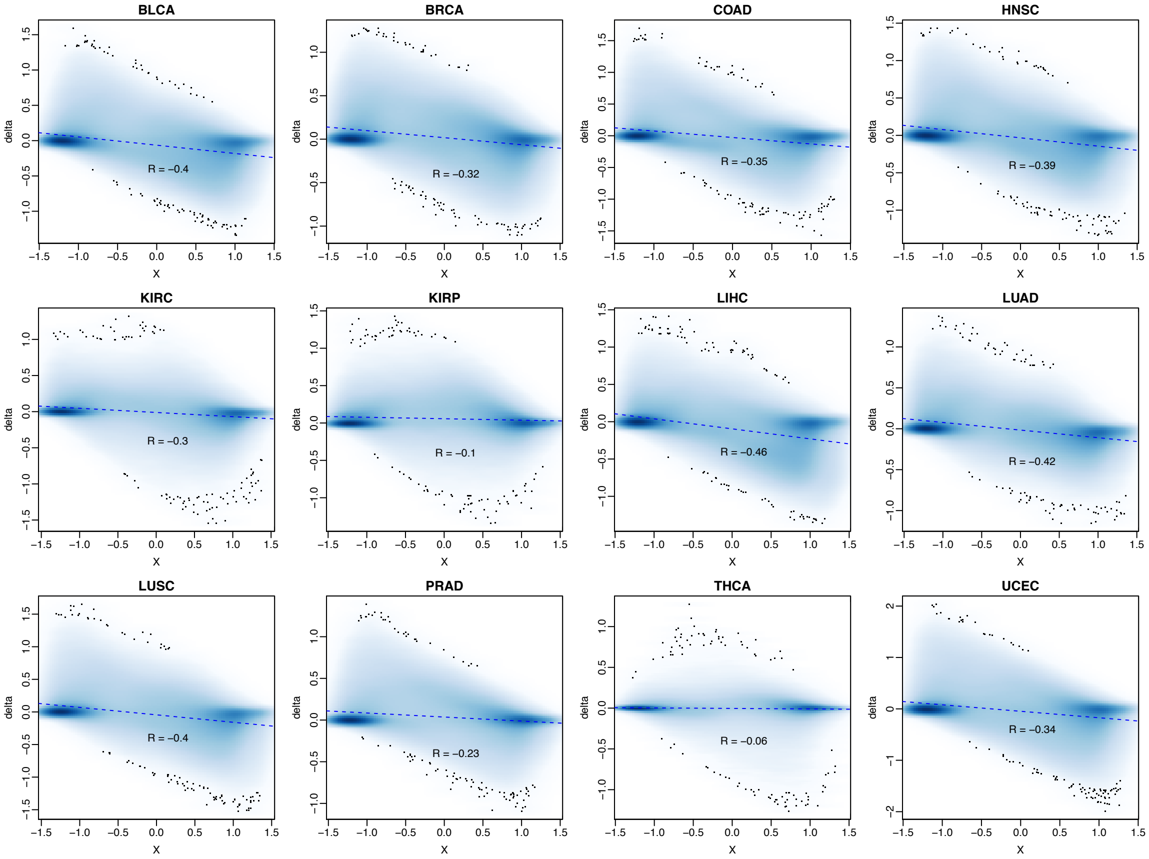

Supplement: Additional file 2: Figures S1 – S18. — (PDF 10334 kb) [file 13059_2016_1143_MOESM2_ESM.pdf]
