# Supplementary material for: Estimating and accounting for tumor purity in the analysis of DNA methylation data from cancer studies
Source: Genome Biol. 2017 Jan 25;18:17. doi: 10.1186/s13059-016-1143-5 (PMC5267453; doi:10.1186/s13059-016-1143-5)
Supplement: Additional file 3: Tables S1 – S4. — (PDF 75 kb) [file 13059_2016_1143_MOESM3_ESM.pdf]

Supplemental Tables for "Estimating and accounting for tumor purity in the analysis of DNA methylation data from cancer studies"

Xiaoqi Zheng[1,$,*], Naiqian Zhang[2,$], Hua-Jun Wu[3] and Hao Wu[4,*]

[1] Department of Mathematics, Shanghai Normal University. Shanghai 200234, China

[2] Department of Mathematics, Weifang University. Weifang, Shandong 261061, China

[3] Department of Biostatistics and Computational Biology, Dana-Farber Cancer Institute and Harvard School of Public Health, Boston 02215, MA, USA

[4] Department of Biostatistics and Bioinformatics, Rollins School of Public Health, Emory University. 1518 Clifton Road. Atlanta, Georgia 30322, USA

[$] These authors contributed equally to this work.

* To whom correspondence should be addressed. Tel: +1(404) 727-8633; Fax: (404)727-1370; Email: hao.wu@emory.edu (HW) or xqzheng@shnu.edu.cn (XZ)

Supplemental Table 1. Correlation of purities with different number of iDMCs for Lung adenocarcinoma.

# iDMCs	Hyper-iDMCs	Hypo-iDMCs	Correlation
40000	19541	20459	-0.029561282
30000	14917	15083	0.075654013
20000	10221	9779	0.269719866
15000	7748	7252	0.395011163
10000	5272	4728	0.47442487
5000	2710	2290	0.633087253
3000	1634	1366	0.732679103
1000	498	502	0.737323616
500	232	268	0.718119456
200	76	124	0.735658178
100	33	67	0.726482289
50	17	33	0.689750702

Supplemental Table 2. 43 universal normal samples used for iDMC detection.

Cancer type	Sample name	Cancer type	Sample name
BLCA	TCGA-CU-A0YN-11A-11D-A10W-05	LUAD	TCGA-49-4488-11A-01D-1756-05
BLCA	TCGA-BT-A20R-11A-11D-A16P-05	LUSC	TCGA-18-5592-11A-11D-1633-05
BRCA	TCGA-BH-A0BM-11A-12D-A093-05	LUSC	TCGA-39-5019-11A-01D-1818-05
BRCA	TCGA-BH-A0DQ-11A-12D-A10Q-05	PAAD	TCGA-FZ-5924-11A-01D-1744-05
CESC	TCGA-MY-A5BF-11A-11D-A26S-05	PAAD	TCGA-H6-A45N-11A-12D-A26Q-05
CESC	TCGA-HM-A3JJ-11A-12D-A21R-05	PCPG	TCGA-P8-A5KC-11A-11D-A35E-05
CHOL	TCGA-W5-AA2I-11A-11D-A418-05	PCPG	TCGA-SQ-A6I4-11A-11D-A35J-05
CHOL	TCGA-W5-AA2U-11A-11D-A418-05	PRAD	TCGA-G9-6385-11A-01D-1787-05
COAD	TCGA-A6-5667-11A-01D-1721-05	PRAD	TCGA-G9-6353-11A-02D-1963-05
COAD	TCGA-G4-6322-11A-01D-1721-05	SARC	TCGA-K1-A42X-11A-11D-A24W-05
ESCA	TCGA-L5-A4OG-11A-12D-A265-05	SARC	TCGA-KF-A41W-11A-11D-A24W-05
ESCA	TCGA-L5-A4OM-11A-11D-A265-05	SKCM	TCGA-FW-A3R5-11A-11D-A23D-05
GBM	TCGA-74-6573-11A-02D-1844-05	SKCM	TCGA-GN-A4U8-11A-11D-A32S-05
HNSC	TCGA-CV-6938-11A-01D-1913-05	STAD	TCGA-BR-6452-11A-01D-1801-05
HNSC	TCGA-CV-6955-11A-01D-2014-05	STAD	TCGA-BR-6566-11A-01D-1801-05
KIRC	TCGA-B0-4718-11A-01D-1275-05	THCA	TCGA-ET-A25N-11A-01D-A16P-05
KIRC	TCGA-BP-5186-11A-01D-1424-05	THCA	TCGA-ET-A3DW-11A-11D-A19K-05
KIRP	TCGA-BQ-7059-11A-01D-1963-05	THYM	TCGA-X7-A8D6-11A-22D-A424-05
KIRP	TCGA-DZ-6135-11A-01D-1963-05	THYM	TCGA-X7-A8D7-11A-11D-A424-05
LIHC	TCGA-FV-A23B-11A-11D-A16X-05	UCEC	TCGA-AJ-A2QL-11A-11D-A18O-05
LIHC	TCGA-FV-A2QR-11A-11D-A20Z-05	UCEC	TCGA-AX-A05Y-11A-11D-A27Y-05
LUAD	TCGA-44-5643-11A-01D-1626-05		

Supplemental Table 3. Overlap of iDMCs and purity correlations by using rank-sum test and minfi for iDMC selections.

Tumor type	iDMC overlap	Purity correlation
ACC	694	0.89916106
BLCA	436	0.9801305
BRCA	429	0.967482834
CESC	591	0.8195898
CHOL	674	0.994111317
COAD	532	0.978500053
DLBC	384	0.835985303
ESCA	629	0.981438551
GBM	613	0.993820524
HNSC	419	0.929794601
KICH	731	0.998078951
KIRC	566	0.834438077
KIRP	643	0.744165123
LAML	677	0.632562598
LGG	550	0.98353618
LIHC	354	0.932238142
LUAD	554	0.938765139
LUSC	501	0.858121655
MESO	749	0.997121086
OV	328	0.99597614
PAAD	599	0.992849575
PCPG	652	0.996418801
PRAD	375	0.642711869
SARC	593	0.336364114
SKCM	512	0.971472702
STAD	644	0.964136807
TGCT	362	0.819077631
THCA	612	0.996071218
THYM	739	0.984293781
UCEC	664	0.99166301
UCS	710	0.996487167
UVM	702	0.996369881

Supplemental Table 4. 32 cancer types analyzed in this study.

Abbr.	Cancer type
ACC	Adrenocortical carcinoma
BLCA	Bladder Urothelial Carcinoma
BRCA	Breast invasive carcinoma
CESC	Cervical squamous cell carcinoma and endocervical adenocarcinoma
CHOL	Cholangiocarcinoma
COAD	Colon adenocarcinoma
DLBC	Lymphoid Neoplasm Diffuse Large B-cell Lymphoma
ESCA	Esophageal carcinoma
GBM	Glioblastoma multiforme
HNSC	Head and Neck squamous cell carcinoma
KICH	Kidney Chromophobe
KIRC	Kidney renal clear cell carcinoma
KIRP	Kidney renal papillary cell carcinoma
LAML	Acute Myeloid Leukemia
LGG	Brain Lower Grade Glioma
LIHC	Liver hepatocellular carcinoma
LUAD	Lung adenocarcinoma
LUSC	Lung squamous cell carcinoma
MESO	Mesothelioma
OV	Ovarian serous cystadenocarcinoma
PAAD	Pancreatic adenocarcinoma
PCPG	Pheochromocytoma and Paraganglioma
PRAD	Prostate adenocarcinoma
SARC	Sarcoma
SKCM	Skin Cutaneous Melanoma
STAD	Stomach adenocarcinoma
TGCT	Testicular Germ Cell Tumors
THCA	Thyroid carcinoma
THYM	Thymoma
UCEC	Uterine Corpus Endometrial Carcinoma
UCS	Uterine Carcinosarcoma
UVM	Uveal Melanoma
